# Resource sharing of an infant gut microbiota synthetic community in combinations of human milk oligosaccharides

**DOI:** 10.1093/ismejo/wrae209

**Published:** 2024-10-18

**Authors:** Athanasia Ioannou, Maryse D Berkhout, William T Scott, Bernadet Blijenberg, Sjef Boeren, Marko Mank, Jan Knol, Clara Belzer

**Affiliations:** Laboratory of Microbiology, Wageningen University & Research, Stippeneng 4, Wageningen 6708WE, the Netherlands; Laboratory of Microbiology, Wageningen University & Research, Stippeneng 4, Wageningen 6708WE, the Netherlands; Laboratory of Systems and Synthetic Biology, Wageningen University & Research, Stippeneng 4, Wageningen 6708WE, the Netherlands; UNLOCK, Wageningen University & Research and Delft University of Technology, Stippeneng 2, Wageningen 6708WE, the Netherlands; Danone Nutricia Research, Uppsalalaan 12, Utrecht 3584CT, the Netherlands; Laboratory of Biochemistry, Wageningen University & Research, Stippeneng 4, Wageningen 6708WE, the Netherlands; Danone Nutricia Research, Uppsalalaan 12, Utrecht 3584CT, the Netherlands; Danone Nutricia Research, Uppsalalaan 12, Utrecht 3584CT, the Netherlands; Laboratory of Microbiology, Wageningen University & Research, Stippeneng 4, Wageningen 6708WE, the Netherlands

**Keywords:** infant gut microbiota, human milk oligosaccharides, synthetic community, community dynamics, bifidobacteria, genome-scale metabolic modelling

## Abstract

Quickly after birth, the gut microbiota is shaped via species acquisition and resource pressure. Breastmilk, and more specifically, human milk oligosaccharides are a determining factor in the formation of microbial communities and the interactions between bacteria. Prominent human milk oligosaccharide degraders have been rigorously characterized, but it is not known how the gut microbiota is shaped as a complex community. Here, we designed BIG-Syc, a synthetic community of 13 strains from the gut of vaginally born, breastfed infants. BIG-Syc replicated key compositional, metabolic, and proteomic characteristics of the gut microbiota of infants. Upon fermentation of a four and five human milk oligosaccharide mix, BIG-Syc demonstrated different compositional and proteomic profiles, with *Bifidobacterium infantis* and *Bifidobacterium bifidum* suppressing one another. The mix of five human milk oligosaccharides resulted in a more diverse composition with dominance of *B. bifidum*, whereas that with four human milk oligosaccharides supported the dominance of *B. infantis*, in four of six replicates. Reintroduction of bifidobacteria to BIG-Syc led to their engraftment and establishment of their niche. Based on proteomics and genome-scale metabolic models, we reconstructed the carbon source utilization and metabolite and gas production per strain. BIG-Syc demonstrated teamwork as cross-feeders utilized simpler carbohydrates, organic acids, and gases released from human milk oligosaccharide degraders. Collectively, our results showed that human milk oligosaccharides prompt resource-sharing for their complete degradation while leading to a different compositional and functional profile in the community. At the same time, BIG-Syc proved to be an accurate model for the representation of intra-microbe interactions.

## Introduction

The gut microbiota formation is paramount for the development of an infant. Quickly upon birth, the sterile gastrointestinal tract gets inoculated by microorganisms mainly deriving from the mother (feces, vagina, skin, mouth) and the surrounding environment [1], among others. The first inhabitants of the infant gut undergo a series of ecological transitions that lead to the establishment of the resident microbial communities. For their carbon, energy, and nitrogen sources bacteria rely on host sources such as mucin and urea but mainly on the non-digestible fraction of breast milk [2].

Breast milk contains not only nutritious compounds that are absorbed by the intestinal epithelium, such as lactose, but also non-digestible carbohydrates. The latter are human milk oligosaccharides (HMOs), glycans with a lactose core that gets elongated with galactose (Gal), N-acetylglucosamine (GlcNAc), fucose (Fuc), or N-acetyl-neuraminic acid (sialic acid, Neu5Ac). These mere five monosaccharides result in more than 200 structures of HMOs [3, 4]. Each woman’s HMO profile is unique and depends on genetic factors such as the secretor status, the gestational age of the infant, and the course of lactation [3]. Some HMOs like 2’-FL, TF-LNH, DF-LNH II, LNFP I, 3’-SL, LNT, 6’-SL, DS-LNT, and 3-FL are very abundant in human milk [5]. These non-digestible carbohydrates can be directly utilized by certain bacteria in the colon. There are indications that this is the major resource pressure that creates interactions between bacteria leading to the establishment of complex microbial communities in the newborn gut [6–8].

The healthy infant gut microbiota is known to be dominated by HMO utilizing bacteria, predominantly the *Bifidobacterium* species. For years, research has focused on studying the beneficial effects of bifidobacteria on infants (e.g. their association with a lower colon pH [9]). However, there is more and more evidence that there are additional pieces in this puzzle. For example, *Bacteroides* spp., are also commonly found in breastfed infants and are positively associated with vaginal delivery [10]. Other, less dominant, bacteria are also present, and their role in the formation and stability of microbial communities has not yet been fully elucidated.

Tracking the ecological relationships between bacteria in the infant gut becomes even more complex when specific species or strains are taken into consideration. Recently, it was shown that bifidobacterial species can outcompete each other and persist also depending on the timing of inoculation [8]. This is also visible at strain level. The infant gut gets inoculated by a plethora of bacteria, some of which are different strains of the same species. As time progresses, it has been suggested that one strain of the same species becomes dominant over the others, thus resembling the adult profile [1]. This is especially important to consider, as certain metabolic capacities are strain-dependent. In the case of HMO degradation, the strain status of a species determines its ability to degrade and utilize certain HMOs [11–14].

HMO degraders have a competitive advantage over other aspiring colonizers of the infant gut. But how do the rest of the bacterial inhabitants manage to secure a place in this highly competitive environment? There are indications that other species can scavenge some HMOs and by-products of HMO degradation. For example, *Lactobacillus casei* BL23 can utilize LNT [15], and many others can grow on leftover lactose and monosaccharides [14, 16, 17]. *Ruminococcus gnavus* can cleave fucose from 2’-FL leaving the lactose free for other bacteria such as *Bifidobacterium breve*, to use [6]. Organic acids can also be cross-fed to other species, such as lactate which is predominantly used by *Veillonella* spp*.* [18]. Others, also possess the Wood–Ljungdahl pathway that enables them to use CO_2_ and H_2_ created by other species in the ecosystem [19]. These results outline the possibility of a collaborative full degradation of HMOs in the gut as driving force of community formation. However, it is not completely clear how infant gut bacterial communities are formed and what the role of each species is, especially in the presence of different HMOs.

Here, we created BIG-Syc, a synthetic community comprising strain-specific bacteria commonly found in the gut of vaginally born breastfed infants. We subjected BIG-Syc to continuous fermentations with different HMO mixes and we retrieved compositional, metabolic, and proteomic profiles similar to those from infant feces. We were able to unravel the mechanisms of HMO degradation in the community and the subsequent resource-sharing that sustains it. Our main findings indicate that different HMO combinations sustain BIG-Syc with alternate predominant HMO degraders dominating in each condition and with bifidobacteria in a special claimable niche.

## Materials & methods

### Anaerobic fermentations of the synthetic community

#### Medium composition and strains used

The strains selected for the synthetic community were either derived from in-house culture libraries or purchased from DSM and ATCC. The complete list of strains includes 13 strains with the respective 16S rRNA gene copy number in parenthesis: *Bifidobacterium infantis* ATCC15697/JCM 1222/DSM 20088 (4), *Bifidobacterium bifidum* JCM1254/DSM 20082 (1), *B. breve* ATCC15700/DSM20213/JCM 1192 (2), *Bacteroides fragilis* ATCC25285 (5), *Bacteroides vulgatus* ATCC8482 (7), *Bacteroides ovatus* DSM1896/ATCC8483 (5), *Veillonella parvula* ATCC10790/DSM2008 (4), *R. gnavus* ATCC 29149 (5), *Escherichia coli* Κ-12 MG1655 DSM18039 (7), *Lactobacillus rhamnosus* GG (5), *Enterococcus faecalis* ATCC19433/DSM20478 (4), *Streptococcus salivarius* subsp. *thermophilus* strain ATCC19258/DSM20617 (5), *Blautia producta* JCM1471/DSM2950 (5). The minimal medium was based on previous work [20] with addition of 1 g/L yeast extract and the exclusion of Na_2_S.7–9H_2_O.

#### Growth of individual strains in HMOs

Pre-cultures were inoculated as 1% v/v in an anoxic minimal medium with 0.2% w/v 2’-FL, or 4HMO, or 5HMO (Table 1, Supplementary Table 1), or no added carbon source. The medium for *B. producta* was modified by the removal of NaHCO_3_ and the use of 100% N_2_ as headspace. The cultures were incubated at 37°C and sampled at 0, 8, 24, 48, and 72 h.

**Table 1:** Specifications of the HMO mixes.

Name	Composition	Purity	Production
2′-FL	2′-FL: 2′-fucosyllactose	>90% DW	Produced using a biotechnological approach
4HMO	2′-FL: 2′-fucosyllactose 39,2 ± 5% DW, 3-FL: 3-fucosyllactose 23,5 ± 3% DW, 3′-SL: 3′-siallylactose 9,8 ± 3% DW, 6′-SL: 6′-siallylactose 27,5 ± 3% DW	>90% DW	Jennewein Biotechnologie GmbH, Rheinbreitbach, Germany
5HMO	2′-FL: 2′-fucosyllactose 52 ± 5% DW, 3-FL: 3-fucosyllactose 13 ± 3% DW, 3′-SL: 3′-siallylactose 4 ± 1% DW, 6′-SL: 6′-siallylactose 5 ± 1% DW, LNT: Lacto-N-Tetraose 26 ± 3% DW	>90% DW	Jennewein Biotechnologie GmbH, Rheinbreitbach, Germany

#### Continuous fermentations in bioreactors

Continuous bioreactors of the DASGIP Parallel Bioreactor Systems (Eppendorf SE, Hamburg, Germany) were set in triplicate. Basal medium with 0.4% w/v 4HMO or 5HMO mix was used. Each strain was inoculated as 1% v/v of an OD:1 normalized culture. Anoxic conditions were monitored based on the redox potential that was constantly below −300 mV. The working volume was 300 ml and the temperature was set to 37°C. The pH was monitored and maintained at 6 with the addition of HCl or NaOH. A mixed gas of 80% N_2_: 20% CO_2_ was introduced at a 6 sl/h rate. The inoculation time was considered to be t0. At the transition to stationary phase based on OD600nm, the fermentations were switched to continuous by introducing fresh medium and removing liquid culture. A flow rate of 12.5 ml/h ensured complete refresh of the medium within 24 h. The bioreactors were sampled at t0, at the last timepoint of the batch fermentation (t19B, t21B, t18B, t19B), and during continuous fermentation at 24 h intervals (t24C, t48C, t72C/t76C, t96C, t120C/t119C/t116C, t146C). For the experiment with species deletion, *Bifidobacterium* spp. were inoculated 73 h into the continuous fermentation.

#### High-performance liquid chromatography

Metabolites were separated with the Shimadzu LC_2030C plus featuring a Shodex SH1821 column and detected via a refractive index detector. The pump supplied the 0.01 N H_2_SO_4_ eluent with a rate of 1 ml/min. The oven temperature was 45°C. The injection volume was 10 μl and the run time 20 min. The internal standard was 10 mM DMSO in 0.01 N H_2_SO_4_ or 0.01 N H_2_SO_4_ and was automatically injected at a 10 μl volume. The external standards were acetate, lactate, propionate, butyrate, iso-butyrate, valerate, iso-valerate, ethanol, glucose, galactose, lactose, GlcNAc, Fucose, Neu5Ac, 1,2-propanediol (1,2-PDO), 1-propanol, 2-propanol, glycerol, succinate, formate, and fumarate. For the monoculture growth in HMOs, 2’-FL, 3-FL, 3’-SL, 6’-SL, and LNT were used as internal standards. Peak annotation and further acquisition of results were performed in Chromeleon X (ThermoFisher Scientific, Massachusetts, USA).

#### HMO extraction and identification

1 ml of culture was processed as in previous work [21]. HMOs present in the samples were identified and quantified based on the LC-ESI-MS^2^ method described previously [22]. We used the latter approach as in previous work [21] with minor adaptations. More specifically, 5 μl of this extract was injected into a 1200/1290 HPLC and in the MS the decrease of solvent A in 0.5 min to 2.5% was kept for 4 min. Samples were analyzed in duplicate.

#### Metaproteomics

Cells from the continuous fermentations were pelleted by centrifugation at 5000 xg, washed with Tris HCl pH 8100 mM, and resuspended in Tris HCl pH 8100 M with Pierce Protease Inhibitor Mini Tablets EDTA free (ThermoFisher Scientific). The cell suspension was sonicated with a cup sonicator (Qsonica, Connecticut, USA) at 4°C using 60 cycles of 15 s ON and 15 s OFF at 100% amplitude. The lysate was reduced by the addition of DL-dithiothreitol reaching a concentration of 13.6 mM. Samples were incubated at 45°C for 30 min and immediately cooled afterwards. Protein unfolding was performed by the addition of 2 times the volume of the sample of 8 M urea. Subsequently, acrylamide was added for protein alkylation, reaching a concentration of 18.2 mM. Samples were then filtered through 70% v/v ethanol pre-washed Nanosep 10 K Omega (Pall Corporation, New York, USA) centrifugal devices for at least 45 min at 13523 xg at 4°C. The protein on the filter was first washed with 2 M ammonium bicarbonate and centrifuged for at least 45 min at 1352 xg at 4°C. It was then washed with 70% v/v ethanol and centrifuged for at least 45 min at 13523 xg at 4°C. The filter was transferred to a new Protein LoBind tube (Eppendorf) and 100 μl 5 ng/μl trypsin in 50 mM ammonium bicarbonate was added. After 1 h incubation at room temperature with shaking, another 10 μl 50 ng/μl trypsin in 50 mM ammonium bicarbonate was added for improved digestion. After overnight incubation at room temperature while shaking, the digesta were centrifuged for 30 min at 13523 xg at 4°C. To completely elute the peptides from the filter, another 100 μl 1 ml/L formic acid in water was added and they were centrifuged for 30 min at 13523 xg at 4°C. Adjustment to approximately pH 3 was achieved by the addition of 3 μl 10% v/v trifluoro-acetic acid. The peptide solution was condensed using a Concentrator Plus (Eppendorf) at 45°C. Subsequently, all samples were reconstituted to 50 μl using 1 ml/L formic acid. Samples were stored at −20°C until further use.

3 μl of peptide samples were subjected to nLC-MS/MS in a reversed-phase nano LC Thermo Vanquish Neo (ThermoFisher Scientific) with in-house capillary columns (1.9 μm particles) coupled to an Orbitrap Exploris 480 (ThermoFisher Scientific), as explained before [23]. An electrospray potential of 3.5 kV was applied.

#### 16S rRNA gene amplicon sequencing & quantitative polymerase chain reaction (qPCR)

1 ml of culture was centrifuged for 5 min at 21300 xg to pellet the cells. DNA isolation, 16S rRNA gene amplicon library preparation of the V5-V6 region (Supplementary Table 2), and sequencing were performed as explained previously [24].

qPCR primers targeting the 16S rRNA gene region were used (Supplementary Table 2). The primers were tested for cross-amplification before the experimental process. The qPCR mix was prepared as shown before [24]. For the enumeration of total bacteria, the cycling program consisted of 1 cycle at 95°C for 3 min, 40 cycles at 95°C for 15 s, then at 52°C for 30 s, and at 72°C for 30 s, and finally a melt curve. For amplification of *Bifidobacterium* spp. and *Bacteroides* spp., the cycling program consisted of 1 cycle at 94°C for 5 min, 40 cycles at 94°C for 20 s, then at 55°C for 20 s, and at 72°C for 50 s, and finally a melt curve.

#### Bioinformatics and data analysis

Data analysis was performed in R (version 4.2.1 [25]) with Rstudio (version 2022.12.0 + 353 [26]), unless otherwise stated.

#### *In silico* investigation of the HMO degrading capacity of publicly available infant gut microbiota metagenomes

Representative sequences per EC number (Supplementary Table 3) were used as queries for BLASTp (command line version 2.11.0) against the publicly available MAGs [27] filtered for infant fecal metagenomes [28]. The results were filtered for <1E-15 e-value and the presence of a GH domain, previously predicted [29], in the same amino acid sequence locus.

#### 16S rRNA gene amplicon sequencing bioinformatics analysis

The quality of the sequencing results was assessed using the FASTQC software [30]. ASV creation and taxonomic assignment were performed with the NG-Tax 2.0 pipeline [31] with default settings using a 100 bp read length and the SILVA138 database. The sequences of the unidentified ASVs were retrieved from the turtle file generated with the NGTax run, queried with BLASTn, and manually assigned as previously [32].

The resulting ASVs were further analyzed using packages phyloseq (version 1.42.0 [33]) and vegan (version 2.6–4 [34]). The ggplot2 (version 3.4.1 [35]) and MicroViz (version 0.10.8 [36]) packages were used for visualization. Core microbiome (detection >0.001 and prevalence >0.5) overlap with publicly available data from infant fecal samples [37] was done with the microbiome (version 1.20.0 [38]) package.

#### Correction of 16S rRNA gene amplicon counts

The raw qPCR data were inspected for efficiency and *R*^2^ and they were extracted with the CFX Manager Software (Bio-Rad Laboratories, California, USA). For species of the same genus, relative abundance per species was inferred as a ratio of the qPCR inferred copy number multiplied by the genus’ relative abundance. The aforementioned results were extrapolated into cell numbers per ml of culture, by multiplying the relative abundance of each species with the 16S rRNA gene copy number of total bacteria divided by the species expected copy number [39].

#### Metaproteomics data analysis

The resulting MS/MS spectra were matched to expected spectra using the Maxquant software (version 2.0.3.0 [40]) with the Andromeda search engine [41]. Fixed modifications were set to “AcrylAmide (C)” and variable modifications to “Oxidation (M)”, “Acetyl (Protein N-term)”, and “Deamidation (NQ)”. All other Andromeda settings were set on default. The protein databases used included the proteomes of the strains in BIG-Syc: *B. fragilis* ATCC25285 (UP000006731), *B. ovatus* ATCC8483 (UP000005475), *B. vulgatus* ATCC8482 (UP000002861), *B. bifidum* (UP000070092), *B. breve* JCM1192 (UP000003191), *B. infantis* ATCC15697 (UP000001360), *B. producta* (UP000515789), *E. faecalis* TX2141 (UP000005586), *E. coli* K12 (UP000000625), *L. rhamnosus* GG (UP000002067), *R. gnavus* ATCC29149 (UP000004410), *Streptococcus salivarius thermophilus* ATCC19258 (UP000249634), *V. parvula* DSM2008 (UP000007968), and *Saccharomyces cerevisiae* (UP000002311) due to the addition of yeast extract in the medium. Common contaminants like Trypsins (P00760, bovin and P00761, porcin) and human keratins (Keratin K22E (P35908), Keratin K1C9 (P35527), Keratin K2C1 (P04264), and Keratin K1CI (P35527)) were included as the contaminants database. Filtering was performed as described previously [23]. nLC-MSMS system and data quality was checked with PTXQC [42].

KEGG Orthologs (KOs) were predicted with KofamKOALA (version 2023-04-01; KEGG release 106.0) [43]. Proteomes were scanned with InterproScan (version 5.62–94.0) [44]) for protein family and pathway annotation.

Statistical testing was performed with Perseus (version 1.6.2.1 [45]). After quality control, contaminant proteins were removed. LFQ intensities were transformed to log10(x) and filtered based on their presence at least twice per condition. Missing values were imputed using “Replace missing values from normal distribution” with default settings. A two-sample t-test was performed with FDR = 0.01 and S0 = 1 as previously [21]. Multiple sample testing with ANOVA (FDR 0.05) and subsequent filtering of significant results was thereon transformed to z-scores. Spearman correlation and average linkage were used for hierarchical clustering.

Proteins were mapped into modules based on their KO assignment as described previously [46] with the curated GMM database.

KOs present in BIG-Syc were compared with bacterial KOs from the EIBER study [47]. The protein identifiers were filtered to remove human and bovine proteins and were searched against UniProt (release 2023_04 [48]) to retrieve their protein sequences. The latter were subjected to KofamKOALA (version 2023-09-07, KEGG release 107.0) to assign KOs (Supplementary Table 4). Statistics of overlap were computed using the “VennDiagram” package (version 1.7.3).

Reconstruction of metabolism was performed by mapping KO information through the KEGG mapper reconstruct tool and by manually searching for enzymes related to HMO degradation [29, 49] and subsequent metabolic pathways.

#### Data analysis of HMO determination

The chromatograms were processed with the software Analyst (version 1.6.2, Sciex, MA, USA). Values were corrected based on the alpha-arabinopentaose standard and duplicates of each fermentor were averaged as previously [21]. The degradation percentage of HMOs and Hex2/lactose were calculated as previously [50].

#### Genome-scale metabolic Modeling analysis

Draft genome-scale models (GEMs) for the 13 target strains were constructed using CarveMe [51], involving the assembly of metabolic pathways and gene-protein-reaction associations based on genomic data. The models were curated to focus on HMO degradation, integrating literature data and refining metabolic pathways employing CobraPy [52]. To ensure model accuracy, flux balance analysis (FBA), flux variability analysis (FVA), and parsimonious FBA were performed, predicting metabolite flow and assessing metabolic network flexibility. Qualitative checks were performed in conjunction with MEMOTE [53] tests to complement quantitative analyses. This involved conducting manual pathway inspections and cross-referencing model predictions with established metabolomics data, to assess discrepancies between predicted and known metabolite production profiles (Supplementary Materials & Methods).

Additional details of our methods can be found in the Supplementary Material. Compilation of multifaceted graphs and minor aesthetic adaptations were performed in Adobe Illustrator (April 2024 release, version 28.5).

## Results

### Assembling a representative synthetic community comprising HMO degraders, primary cross-feeders, and acetogens

**Figure 1:**
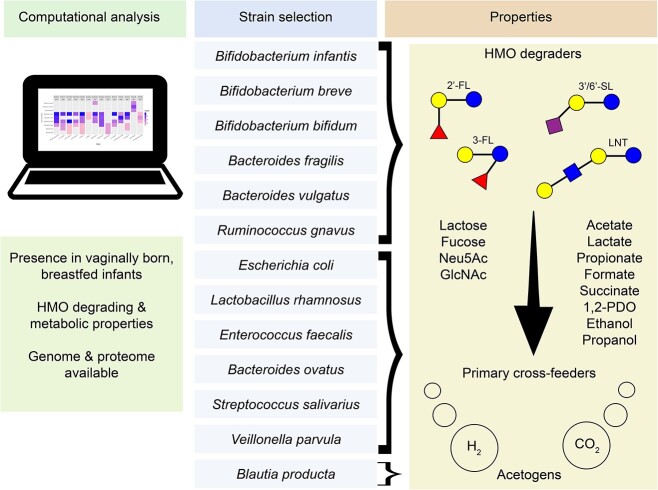
Assembly of the synthetic community based on computational analysis to select for the strains with desired characteristics. The 13 strains are predicted to belong to the trophic levels of HMO degraders, primary cross-feeders and acetogens.

The composition of BIG-Syc (**B**acterial **I**nfant **G**ut **Sy**nthetic **C**ommunity) was selected based on the 20 most prevalent and most abundant taxa found in 4-month-old vaginally born infants [28] (Supplementary Table 5, Supplementary Fig. 1). Specific strains were selected based on the most up to date literature for their presence in infant gut, their metabolic characteristics, and data availability (publicly available full genomic sequence, publicly available complete proteome database, 16S rRNA gene copy number information). The HMO-degrading potential of the selected genera/species was further researched in publicly available Metagenome Assembled Genomes (MAGs) [27] (Supplementary Figs. 2–3). This search was based on glycoside hydrolase (GH) families that are expected to take part in degradation of HMOs, previously reviewed by us [29]. Focusing on a Bottom-Up approach we initially grew each of the selected strains in basal medium with 2’-FL, 4HMO, 5HMO, and no carbon source to identify the HMO degraders (Supplementary Fig. 4, Fig. 1). This is expected to lead to the release of simpler sugars, organic acids, and gases. Primary cross-feeders can utilize sugars and organic acids, whereas acetogens can use the gases produced.

### Shift of bifidobacterial relative abundance in the added presence of LNT

**Figure 2:**
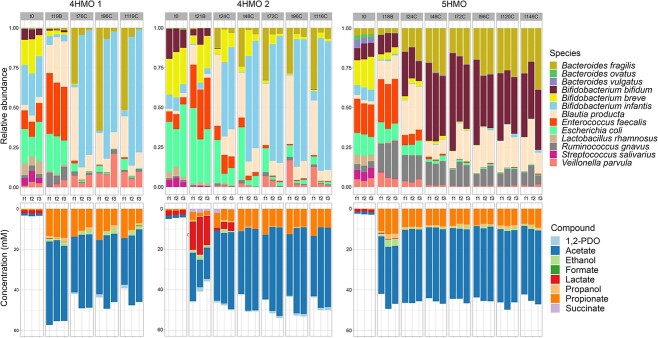
Top: Composition plots of qPCR and copy number corrected relative abundance of species per fermentor, grouped per timepoint. Bottom: Composition plots of the concentration (mM) of metabolites per fermentor, grouped per timepoint.

The mix of selected strains was subjected to continuous fermentation with HMOs as sole carbon source. With this, BIG-Syc was established after 48 h (t48C) to 72 h (t72C) of continuous fermentation (Fig. 2, Supplementary Table 6). The 4HMO condition was performed twice (4HMO 1 and 4HMO 2), due to high variability between replicates, and the 5HMO once (5HMO). The synthetic community remained highly pure (>99.1% mean relative abundance of community members) in both the 4HMO and 5HMO conditions, as verified by qPCR and copy number corrected 16S rRNA gene amplicon sequencing (Supplementary Table 7). As time progressed, HMO degraders thrived in the community and allowed for the growth of non-HMO degraders reaching an average of 3.47E+10 (SD: ±1.74E+10) 16S rRNA gene copies. For the 4HMO condition, where LNT is not present, *B. infantis* dominated the community in two fermentors but was suppressed in the other fermentor giving space to *B. fragilis* and *B. producta*. In the 5HMO condition, *B. bifidum* and *B. fragilis* dominated the community together with *B. producta*. Composition-wise, for both experiments with 4HMO, fermentors 2 and 3 were more similar to one another than with fermentor 1 based on Spearman correlation coefficient (Supplementary Fig. 5A). For the 5HMO condition, fermentors had a higher Spearman correlation coefficient in all timepoints. In all conditions, BIG-Syc produced mainly acetate and propionate with smaller amounts of ethanol, propanol, and 1,2-PDO (Fig. 2). Lactate and succinate were produced at earlier timepoints and were mostly depleted by the final timepoint of the fermentation. At the final measurement points, the metabolic profiles between fermenters showed a Spearman correlation coefficient greater than 0.9, except for the profiles related to 4HMO 1 in fermentor 1 (Supplementary Fig. 5B).

### BIG-Syc in minimal medium with HMOs captures the compositional and functional profiles of pre-weaned infants

**Figure 3:**
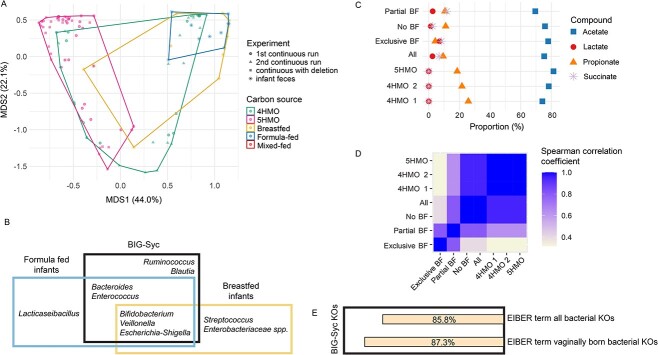
A) PCoA plot of the bray–Curtis dissimilarity calculated for the 16S rRNA gene amplicon data of <5 month old infants and BIG-Syc in continuous fermentations, B) Venn diagram of shared core genera between breastfed infants, formula fed infants, and the 4HMO and 5HMO continuous fermentations, C) relative abundance of mean acetate, propionate, lactate, and succinate in the sum of total SCFA, lactate, and succinate, D) Pearson correlation coefficient between different samples for relative abundance of mean acetate, propionate, lactate, and succinate in the sum of total SCFA, lactate, and succinate, E) percentage of bacterial KOs from term infant fecal samples of the EIBER study that are also present in BIG-Syc proteomics.

We further examined whether BIG-Syc—grown in minimal medium with HMOs—can serve as a reduced complexity model of the gut microbiota in pre-weaned infants. For this, we extracted 16S rRNA gene amplicon data from our previously published cohort of 21 infants of a maximum of 21 weeks of age [37] and metabolites from a published cohort of infants of 3 to 5 months of age [54]. Convex hulls of Bray–Curtis dissimilarity in PCoA ordination illustrate that infant gut microbial communities overlap with BIG-Syc in terms of genus composition and relative abundance (Fig. 3A). Even though BIG-Syc contains nine genera instead of the 76 distinct genera found in infants, there are core genera (detection >0.001 and prevalence >0.5) shared between them (Fig. 3B). All genera identified as core genera for the infant fecal samples are present in the synthetic community. Additionally, *Bifidobacterium* spp., *Veillonella* spp., *Escherichia-Shigella* spp., *Bacteroides* spp., and *Enterococcus* spp. are identified as core genera in both BIG-Syc and fecal samples. *Ruminococcus* spp. and *Blautia* spp. were core genera of BIG-Syc but they were not identified as core genera in infants. In contrast, *Lacticaseibacillus* spp., *Streptococcus* spp. (even though contained in BIG-Syc), and *Enterobacteriaceae* spp. are only identified as core genera in infant fecal samples. This demonstrates that the bacteria present in BIG-Syc are important drivers for gut microbial communities in pre-weaned infants.

In terms of metabolic profiles, BIG-Syc shares acetate, propionate, succinate, and lactate as common compounds with real fecal samples. To allow for the comparison, the percentage relative abundance of mean acetate, propionate, lactate, and succinate in the sum of total SCFA, lactate, and succinate was computed (Fig. 3C). Acetate relative abundances are the closest between BIG-Syc and fecal samples. Spearman correlation coefficient showed a higher correlation (>0.9) between the different HMO conditions, infants with no breastfeeding (no BF), and the total infant pool (all) (Fig. 3D). Lower correlation coefficients were observed with partially breastfed (Partial BF) infants (0.63) and exclusively breastfed (Exclusive BF) infants (0.32). Butyrate, iso-butyrate, valerate, and iso-valerate are not produced by BIG-Syc members and, hence, they were not detected in our experiments. Additionally, succinate and lactate relative abundances are lower because they are utilized as carbon sources by non-HMO degraders.

We also compared the protein functionality of BIG-Syc to that of fecal samples of term infants from the published EIBER study [47]. We mapped the bacterially expressed proteins to KO identifiers and we compared the overlap between BIG-Syc and fecal samples (Fig. 3E). BIG-Syc grown on HMOs covered 85.8% of the KOs in fecal samples of all three term infants and 87.3% of fecal samples from the vaginally born infant.

### Complete HMO degradation relied on specialized strategies of HMO degraders

**Figure 4:**
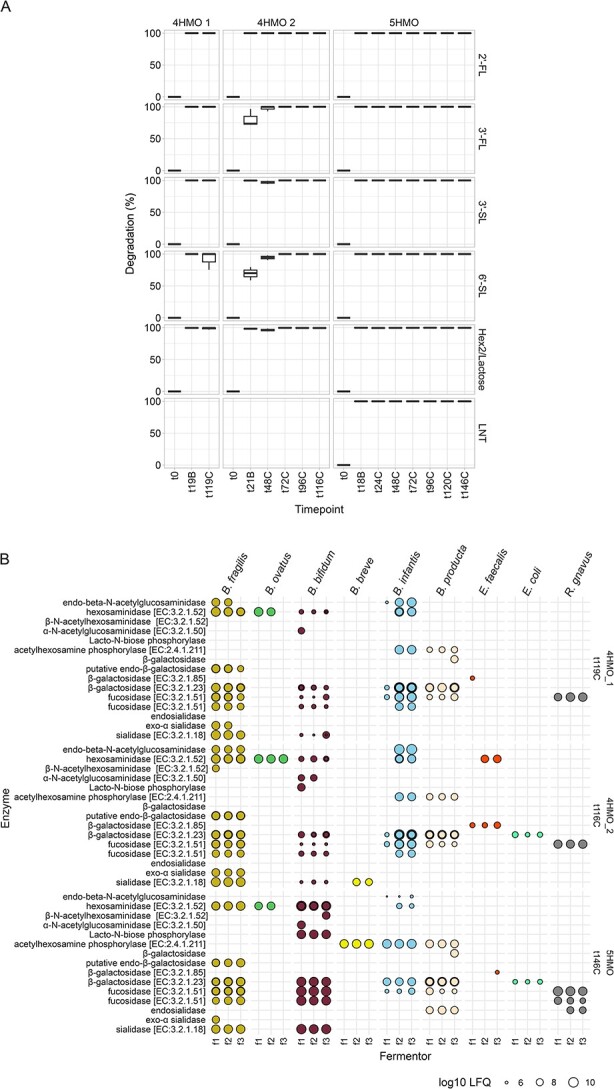
A) Degradation percentage of 2’-FL, 3-FL, 3’-SL, 6’-SL, LNT and Hex2/lactose per timepoint and condition, B) enzymes related to HMO degradation, grouped per species, fermentor, and condition in the final timepoint. Shortcut labels: “1,3-beta-galactosyl-N-acetylhexosamine phosphorylase [EC:2.4.1.211]” = “acetylhexosamine phosphorylase [EC:2.4.1.211]”, “6-phospho-beta-galactosidase [EC:3.2.1.85]” = “β-galactosidase [EC:3.2.1.85]”, “alpha-L-fucosidase [EC:3.2.1.51]” = “fucosidase [EC:3.2.1.51]”, “alpha-L-fucosidase 2 [EC:3.2.1.51]”=“fucosidase [EC:3.2.1.51]”, “alpha-N-acetylglucosaminidase [EC:3.2.1.50]” = “α-N-acetylglucosaminidase [EC:3.2.1.50]”, “beta-galactosidase [EC:3.2.1.23]” = “β-galactosidase [EC:3.2.1.23]”, “beta-N-acetylhexosaminidase [EC:3.2.1.52]” = “β-N-acetylhexosaminidase [EC:3.2.1.52]”, “hexosaminidase [EC:3.2.1.52]” = “hexosaminidase [EC:3.2.1.52]”,"sialidase-1 [EC:3.2.1.18]” = “sialidase [EC:3.2.1.18]”, “putative endo-beta-galactosidase” = “putative endo-β-galactosidase”, “Exo-alpha sialidase” = “exo-α sialidase”, “Endosialidase” = “endosialidase”, “beta-galactosidase” = “β-galactosidase”, “Endo-beta-N-acetylglucosaminidase” = “endo-beta-N-acetylglucosaminidase”, “Lacto-N-biose phosphorylase-like N-terminal TIM barrel domain-containing protein (Fragment)”=“lacto-N-biose phosphorylase”.

BIG-Syc was able to fully degrade (>90%) each of the carbohydrate components during continuous fermentation of the 4HMO and the 5HMO mix as measured with LC-ESI-MS^2^ (Fig. 4A). The only exception was 6’-SL at t119C 4HMO 1, where fermentors differed in the degradation percentages. Any remaining lactose from pre-cultures, as well as hexoses and lactose from extra-cellular HMO degradation, were also fully degraded by the community.

To further specify the carbohydrate degradation in BIG-Syc, we performed metaproteomics and focused on a selection of enzymes from our results that are known to be involved in HMO degradation [29]. *B. infantis, B. bifidum*, and *B. fragilis* produced an array of enzymes designated for complete HMO degradation (Fig. 4B). Despite not being able to grow on HMOs as a sole carbon source, *B. producta* produced multiple enzymes with possible HMO degrading capacity. *B. breve* produced sialidases in fermentors 2 and 3 in the 4HMO 2 condition, whereas in the 5HMO condition, it produced a 1,3-beta-galactosyl-N-acetylhexosamine phosphorylase [EC:2.4.1.211]. *R. gnavus* expressed fucosidases in all conditions and an endo-sialidase in the 5HMO degradation.

**Figure 5:**
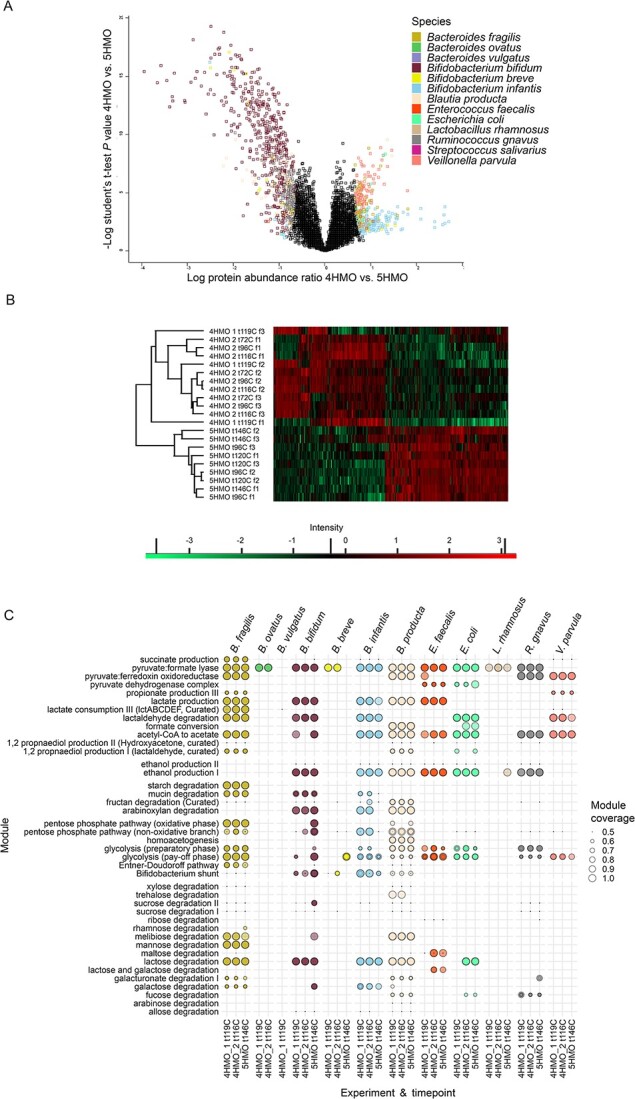
A) Volcano plot of -log t-test *P* value (y axis) and log protein abundance ratio (t-test difference) (x axis) between 4HMO and 5HMO. Significantly different proteins are colored per species and non-significantly different proteins are in black color. B) Heatmap of hierarchical clustering based on spearman correlation of ANOVA determined significantly different proteins. C) Overview of relevant modules with >0.5 KO coverage. Values are grouped per module, experiment, and species in the final timepoint. Values from the three fermentors overlap so lighter color indicates the module was detected in fewer fermentors. Different coverages between fermentors lead to overlapping circles of different sizes. Modules are ordered by their relation to organic acids, alcohols, complex carbohydrates, main pathways and simple carbohydrates.

The volcano plot generated by -log t-test *P* value plotted over the log protein abundance ratio (t-test difference) (Fig. 5A) shows differences in protein expression between the 4HMO and 5HMO conditions. Out of the 6122, 1178 were statistically significantly different between the two conditions (Supplementary Table 8). In the 4HMO experiments, proteins from *B. infantis* (175)*, V. parvula* (112)*, B. fragilis* (44)*,* but also *E. coli* (10)*, B. breve* (9)*, B. producta* (7), *E. faecalis* (6)*, B. ovatus* (2), and *L. rhamnosus* (1) were significantly higher. In contrast, in the 5HMO experiments, proteins from *B. bifidum* (533), *B. producta* (133), and *R. gnavus* (117)*,* and to a lesser extent *B. breve* (12)*, B. infantis* (7)*, B. fragilis* (5), *B. vulgatus* (3), *E. faecalis* (1)*,* and *E. coli* (1) were significantly higher. Hierarchical clustering further demonstrates differential expression of proteins between the two conditions (Fig. 5B). The 5HMO condition was more unanimous in its expression profile, whereas in both runs of the 4HMO condition, fermentor 1 clustered separately from the other fermentors.

Metabolic modules of *B. fragilis, B. bifidum, B. breve, B. infantis, B. producta*, and *R. gnavus* showed they expressed proteins for complex carbohydrate utilization (Fig. 5C, Supplementary Fig. 6). The aforementioned bacteria along with *E. faecalis, E. coli,* and *L. rhamnosus* produced proteins for simple carbohydrate utilization and the glycolysis pathways. *B. ovatus* showed only pyruvate formate-lyase related proteins. *B. vulgatus* and *B. fragilis* together with *V. parvula* proteins are involved in lactate consumption, albeit for the former in only the 4HMO 1 run.

### Bifidobacteria are successfully engrafted after supplementation in BIG-Syc

**Figure 6:**
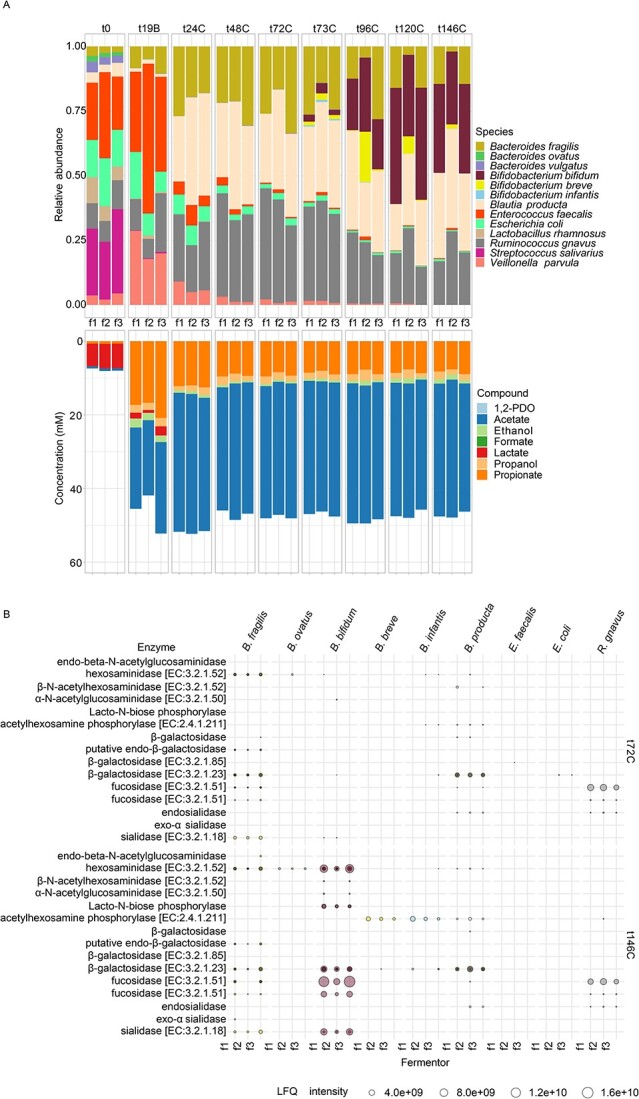
A) Top: Composition plots of qPCR and copy number corrected relative abundance of species per fermentor, grouped per timepoint. Bottom: Composition plots of the concentration (mM) of metabolites per fermentor, grouped per timepoint. B) Enzymes related to HMO degradation, grouped per species and fermentor in the final timepoint before and after addition of bifidobacteria. Shortcut labels: “1,3-beta-galactosyl-N-acetylhexosamine phosphorylase [EC:2.4.1.211]” = “acetylhexosamine phosphorylase [EC:2.4.1.211]”, “6-phospho-beta-galactosidase [EC:3.2.1.85]” = “β-galactosidase [EC:3.2.1.85]”, “alpha-L-fucosidase [EC:3.2.1.51]” = “fucosidase [EC:3.2.1.51]”, “alpha-L-fucosidase 2 [EC:3.2.1.51]”=“fucosidase [EC:3.2.1.51]”, “alpha-N-acetylglucosaminidase [EC:3.2.1.50]” = “α-N-acetylglucosaminidase [EC:3.2.1.50]”, “beta-galactosidase [EC:3.2.1.23]”  = “β-galactosidase [EC:3.2.1.23]”, “beta-N-acetylhexosaminidase [EC:3.2.1.52]” = “β-N-acetylhexosaminidase [EC:3.2.1.52]”, “hexosaminidase [EC:3.2.1.52]” = “hexosaminidase [EC:3.2.1.52]”,"sialidase-1 [EC:3.2.1.18]” = “sialidase [EC:3.2.1.18]”, “putative endo-beta-galactosidase” = “putative endo-β-galactosidase”, “Exo-alpha sialidase” = “exo-α sialidase”, “Endosialidase” = “endosialidase”, “beta-galactosidase” = “β-galactosidase”, “Endo-beta-N-acetylglucosaminidase” = “endo-beta-N-acetylglucosaminidase”, “Lacto-N-biose phosphorylase-like N-terminal TIM barrel domain-containing protein (Fragment)”=“lacto-N-biose phosphorylase”.

To better understand the dependencies in community structure in BIG-Syc, we performed a deletion experiment, using the 5HMO mix as carbon source. All strains apart from *Bifidobacterium* species were inoculated at t0. After 3 days of continuous fermentation (t73C), all three *Bifidobacterium* species were introduced. Three days after addition (t146C), the relative abundance of the genus-level *Bifidobacterium* increased and resembled that of the experiment with the entire synthetic community (Fig. 6A). *B. bifidum*, that reached the highest relative abundance, expressed all the necessary proteins for HMO degradation, i.e. sialidases, fucosidase, hexosaminidases, Lacto-N-biose phosphorylase, and β-galactosidase (Fig. 6B). The expression profile of *B. fragilis* and *R. gnavus* was not restricted with the introduction of bifidobacteria. *B. breve* and *B. infantis* that reached lower relative abundances, produced 1,3-beta-galactosyl-N-acetylhexosamine phosphorylase [EC:2.4.1.211] and β-galactosidase, demonstrating that they probably scavenged by-products of HMO degradation by *B. bifidum* and *B. fragilis*. Proteins from *E. faecalis* and *E. coli* were completely depleted by the final timepoint of the fermentation. The addition of bifidobacteria resulted in a statistically significant increase in ethanol (*P* = 0.005) and a decrease in propionate (*P* = 0.026) but not in acetate, propanol, and 1,2-PDO (Supplementary Fig. 7).

### Community structure and metabolic crossfeeding in HMOs

**Figure 7:**
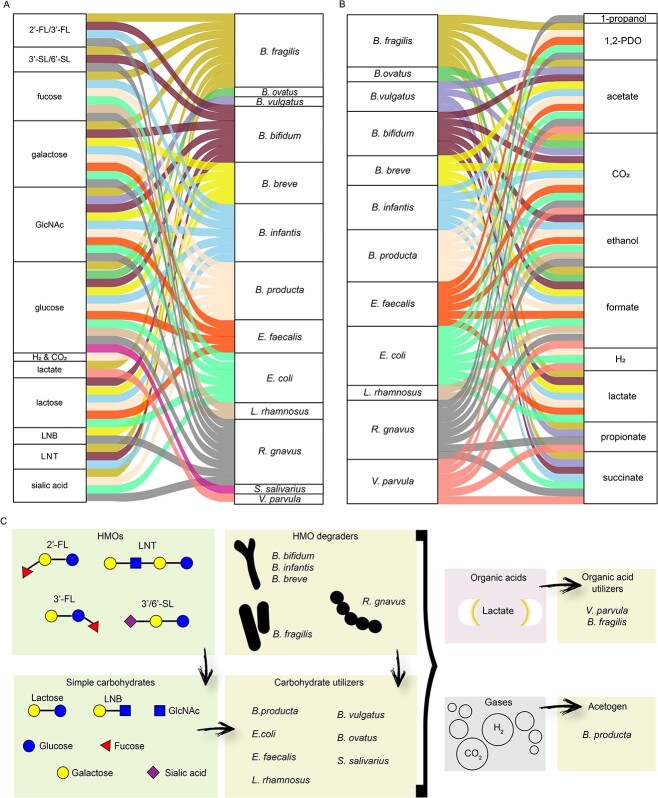
A) Inflow of carbon sources for each BIG-Syc member and B) outflow of metabolites and gases. C) Carbon source utilization strategies and trophic level interactions of BIG-Syc members grown on HMOs.

Metaproteomics and HPLC results from monoculture experiments were used to justify HMO utilization (Fig. 7A, Supplementary Table 9). With metaproteomics results as a backbone, we reconstructed metabolite production (Fig. 7B, Supplementary Table 10) by each BIG-Syc member. Gas production was inferred via metaproteomics and was further corroborated with the use of genome-scale metabolic models curated for the 4HMO and 5HMO mix (Fig. 7B, Supplementary Table 10). We also compared the per-species metabolic outputs predicted from the GEMs with the metabolite profiles inferred from our *in vitro* proteomics data, which did not fully align. The differences between *in silico* and *in vitro* metabolite production, possible reasons behind these inconsistencies, and the implications are presented in the Supplementary Materials & Methods. With this, we were able to reconstruct the resource-sharing in BIG-Syc and to distinguish four types of carbon source utilization strategies (Fig. 7C). Those comprise the HMO-degraders, the simple carbohydrate utilizers, the organic acid utilizers, and the acetogen. The HMO degraders, namely *B. infantis*, *B. bifidum*, *B. breve*, *B. fragilis*, and *R. gnavus* were able to fully degrade the HMOs present, with a preference for some structures (Fig. 7A). The fucosyllactoses were utilized by *B. infantis*, *B. fragilis*, *B bifidum*, and *R. gnavus*, the siallylactoses by *B. fragilis*, *B. bifidum*, and *R. gnavus,* and LNT by *B. fragilis*, *B. bifidum,* and *B. infantis*. The extracellular degradation of the HMOs liberated LNB, lactose, and monosaccharides that were subsequently scavenged by the simple sugar utilizers, i.e. *B. ovatus*, *E. coli*, *E. faecalis*, *L. rhamnosus,* and *S. salivarius*. *B. vulgatus* was only able to grow in very low numbers and did not demonstrate HMO degradation but rather utilization of simpler carbohydrates. Also, the acetogen *B. producta* scavenged simple carbohydrates, H_2_, and CO_2_ from the medium. Lastly, *V. parvula* relied on lactate production by other strains for its carbon source. Acetate, the most abundant organic acid, was produced by all strains apart from *B. ovatus*, *S. salivarius*, and *L. rhamnosus*. Propionate was the secondary metabolite of *B. fragilis*, *B. vulgatus*, *R. gnavus*, and *V. parvula*. Lactate, ethanol, formate, and succinate were also produced by many of the BIG-Syc members. 1,2-PDO, that was mostly measured in the 4HMO condition, was produced by *B. fragilis*, *B. producta*, *E. faecalis*, *E. coli*, and *R. gnavus*, but only the latter was able to transform it to 1-propanol. Many metabolite conversions led to the production of CO_2_ as well as H^+^ that was channeled to H_2_ formation.

## Discussion

Current knowledge about the ecological succession in the infant gut contains gaps as to how the microbiota is formed and sustained when only a few species possess the ability to utilize HMOs. To accurately focus on the trophic interactions between bacteria, we designed BIG-Syc, a 13-strain synthetic community of bacteria commonly found in the gut of vaginally born, breastfed infants. Based on rigorous literature and bioinformatics analysis, we picked specific strains that were expected to fulfill various niches when subjected to continuous fermentations in a minimal medium with HMOs. These are HMO degraders, primary cross-feeders, and acetogens.

Feeding BIG-Syc with a 4HMO and a 5HMO mix resulted in compositional profiles where HMO degraders, specifically bifidobacteria and *B. fragilis*, were dominant in terms of relative abundance in the synthetic community. Our results are in line with two recent studies utilizing co-cultures in batch fermentations [8] and fecal samples of vaginally born infants up to 6 months of age [7] where there is alternate dominance of *B. infantis* and *B. bifidum*/*B. breve*. In the 4HMO condition, *B. infantis* dominated over *B. bifidum* in two of three fermentors in both runs where *B. infantis* reached more than 70% of the relative abundance. We hypothesize that 6’-SL utilization is a determining factor. *B. infantis* ATCC15697 is the most efficient utilizer of 2’-FL based on our monocultures and *B. bifidum* JCM1254 is known to not grow on 6’-SL [13]. *B. bifidum* produced sialidases in 4HMO 2 and 5HMO, however, the SiaBb2 sialidase is mostly active on α-2,3 bonds instead of the α-2,6 bonds holding the 6’-SL structure [55]. We can also see this in our monocultures where the degradation of 3’-SL is much higher than that of 6’-SL. We did not measure any sialidases or enzymes related to sialic acid utilization from *B. infantis*, which restricts it to 2’-FL/3-FL utilization.

In the 5HMO condition, *B. bifidum* suppressed the growth of *B. infantis*. A similar pattern has been previously observed in co-cultures of the same *B. bifidum* and *B. infantis* strains in a mix of highly fucosylated HMOs together with LNT [8]. In the same study, when *B. bifidum*, *B. breve, B. infantis*, and *Bifidobacterium longum* were inoculated together, the first two dominated the community, agreeing with our results. In this condition, LNT is the second most abundant HMO after 2’-FL. LNT contains GlcNAc which, together with its derivatives, was recently found to control NagR, a major regulator of multiple genes and operons in bifidobacteria [56]. It is thus possible that the presence of LNT upregulates HMO utilizing genes in *B. bifidum* making it more competitive in this environment. The fact that *B. bifidum* grows faster in our monocultures when the 5HMO mix is added along with transcriptomic data where LNT may generate fucosidase expression in this species [13] supports this notion. In contrast, *B. infantis* may be less competitive in an environment where LNT is extracellularly cleaved. The LNB-GNB cluster is downregulated when this strain is grown in 2’-FL [57], which has been supported by transcriptomics results showing a different profile for 2’-FL and LNT utilization [13]. Nevertheless, there are still many unknowns on expression profiles by bifidobacteria in the presence of HMOs, a topic that becomes even more complex when different strains are taken into account [13, 58, 59].

Another prominent HMO degrader, *B. breve*, grew in both the 4HMO and, to a lesser extent, the 5HMO condition, and it relied on lactose, galactose, glucose, GlcNAc, and LNB utilization. We will not focus on the ability of *B. breve* to flourish in the presence of *B. bifidum*, as this has been extensively proven to be because of the altruistic ability of *B. bifidum* to extra-cellularly degrade HMOs [17, 55, 60, 61]. A similar interaction has been suggested between *R. gnavus* and *B. breve* where the former releases lactose in the medium [6]. Apart from simpler structures, *B. breve* also uses LNT [58] which makes it a notable inhabitant of the gut of breastfed infants [28] and, thus, was expected to flourish in the 5HMO condition. Its restricted presence in the second run of the 4HMO condition and after reintroduction of the bifidobacteria in the 5HMO deletion experiment signifies that it must compete for simpler carbohydrates. In the 4HMO condition, it also produced a sialidase that is homologous to that of *B. infantis* ATCC15697 [50, 58]. This enzyme enables this strain to grow on bigger acidic HMOs like LST, but not in 3’-SL/6’-SL according to previous literature [58].

We wanted to further understand whether bifidobacteria have a clear niche in BIG-Syc fermenting HMOs, so we introduced them in a deletion experiment. The vast dominance of *B. infantis* in the 4HMO condition, corroborated by supplementation trials [62, 63], led us to choose for the 5HMO condition featuring a more diverse community. Compositional and proteomic results showed that bifidobacteria not only had a claimable niche among other bacteria in an HMO-rich environment, but also that this niche is not affected by priority. The reduction of *B. fragilis*’ relative abundance suggests that *B. bifidum* and *B. breve* compete with it for a similar niche and succeed in overtaking it even when a stable community on the same nutrient source has already been established.

The 6’-SL of the 4HMO mix was probably utilized by *B. fragilis* ATCC25285 that is known to utilize this HMO [59, 64, 65]. In monoculture, *B. fragilis* was not an efficient 2′/3-FL utilizer, however, previous studies have shown it can grow on these HMOs [64]. This strain expressed a wide array of HMO-degrading enzymes, including sialidases that give it a competitive advantage, also against other *Bacteroides* spp. [65]. Current literature on HMO utilization by *Bacteroides* is scarce, but studies so far create the picture of a mucin-like type of degradation that is mostly extracellular [65, 66]. This could explain, together with the growth of *B. bifidum*, the more diverse community supported by the 5HMO condition.

In the 5HMO condition, we saw an increased relative abundance of *R. gnavus*. This strain utilizes 2′−/3-FL, albeit releasing fucose and lactose which cannot be used by this strain [67]. It also possesses an α-2,3-acting trans-sialidase targeting 3’-SL, through which sialic acid is transformed to its anhydrous form [68]. These together with the extracellular localization of its enzymes, make it a pivotal HMO-degrader for the rest of the community. Considering its preference for fucosyllactoses, it can be deduced that in the absence of *B. infantis* in this condition, *R. gnavus* finds its niche by degrading these HMOs.

*B. ovatus*, *B. vulgatus*, *L. rhamnosus*, *S. salivarius*, *E. faecalis*, and *E. coli* proliferated via cross-feeding of simple sugars, nevertheless in low relative abundances. Carbon source pressure and anoxic conditions are possible reasons behind their suppression. In contrast, *V. parvula* efficiently scavenged its preferred carbon source, lactate, especially in the 4HMO condition. *B. fragilis* showed signs of both lactate production and utilization, the latter being still under investigation throughout literature [69, 70]. Intermediate metabolites such as succinate and lactate are quickly utilized by BIG-Syc, whereas they are measurable in infant fecal samples. Other by-products of fermentation that were expected to be assimilated are CO_2_ and H_2_. The acetogen*, B. producta* proved to also utilize simple carbohydrates, which explains its high abundance. *Blautia* expressed fucosidases and sialidases, however, it cannot grow with HMOs as the sole carbon source. This could mean that the enzymes are either not functionally specific for HMOs or that it lacks HMO-specific transporters. Other bacteria, including bifidobacteria, express inefficient HMO degrading enzymes [13] and it would be worth investigating the evolutionary process of their genes.

The ecological relationships of the infant gut microbiota were investigated here using a simplified model that revealed known limitations of such systems. Firstly, we were faced with the inherent need to select certain strains. As HMO degradation is species- or strain-dependent, exchange of a certain strain might have led to a different compositional or functional profile. Additionally, in an effort to accurately portray bacterial metabolism and cross-feeding, we used a minimal medium and predefined HMO mixes. Complex and variable HMOs, non-degraded lactose, mucin glycans, and proteins reaching the infant gut, create an additional complexity in resource pressure. Moreover, the intricate host parameters such as the uptake of metabolites, the immunological crosstalk, and the intermittent feeding, are yet to be replicated *in vitro*. Further utilization of the GEMs and the prediction of interactions between species in the community would require additional manual curation and thorough testing, as we reported inconsistencies compared to our *in vitro* results. Similar challenges have been showcased by other researchers in efforts to improve GEM predictions [71–73]. This highlights the need for manual, detailed, and evidence-based curation of GEMs and FBA to achieve reliable predictions and draw meaningful biological conclusions. Lastly, we also need to mention the variability between replicates which led us to an additional run of the 4HMO condition. Excluding technical variability due to rigorous calibration, this would be explained by “bistability”, the presence of two stable steady states, decided by earlier bacterial interactions [74]. The accretive use of complex microbial communities will provide more scientific evidence for this ecological attribute.

Despite the aforementioned limitations, BIG-Syc proved to be an accurate reduced complexity model. Its similarity with fecal samples, in terms of omics data, which we focus on in this study, increases the confidence that BIG-Syc is indicative of the natural occurrences in the gut of pre-weaned infants. However, the goal of BIG-Syc is not to mirror the infant gut microbiota. This would imply a higher compositional diversity of the synthetic community that would compromise its ability to portray clear ecological relationships between species. Our study shows that synthetic communities can be used as a reliable research model, but also as a means to test food supplements. The establishment of bifidobacteria after introduction at a later stage paves the way for possible treatment strategies to restore the perturbed infant gut microbiota. This could include applications as probiotics or as synbiotics, together with a stimulating compound that is carefully chosen to match the probiotics used. Additionally, we have managed to portray higher-order interactions in the presence of HMOs showing that the formation of the infant gut microbiota in the gut of breastfed infants does not just rely on bifidobacteria. *Bacteroides* spp. are a promising candidate as here they demonstrated HMO degrading capacity and resource sharing. Butyrate producers and/or solid food fibers could allow for the investigation of the maturing infant gut microbiota. Adding other domains of life, such as archaea and viruses, would shed light on the ecological establishment of the gut microbiota, possibly governed by priority effects and resource pressure not currently explained solely by bacteria.

## Supplementary Material

Ioannou_Belzer_supplementary_R3_clean_wrae209

Supplementary_Table_6_wrae209

Supplementary_Table_8_wrae209

## Data Availability

Raw reads from 16S rRNA gene amplicon sequencing are stored at ENA (EMBL-EBI) as study accession number PRJEB72539. Raw files from metaproteomics are stored at the PRIDE database (EMBL-EBI) under the accession number PXD047785. The genome-scale metabolic models are stored at the BioModels database (EMBL-EBI) under the accession number MODEL2405300001. The code to process and visualize the data produced in this study, as well as that for the genome-scale metabolic models can be found at https://git.wur.nl/afsg-microbiology/publication-supplementary-materials/big-syc/big-syc-continuous-fermentations-in-hmos.git.
