# Supplementary material for: Resource sharing of an infant gut microbiota synthetic community in combinations of human milk oligosaccharides
Source: ISME J. 2024 Oct 18;18(1):wrae209. doi: 10.1093/ismejo/wrae209 (PMC11542058; doi:10.1093/ismejo/wrae209)
Supplement: Ioannou_Belzer_supplementary_R3_clean_wrae209 [file ioannou_belzer_supplementary_r3_clean_wrae209.docx]

# Supplementary Material

**Resource sharing of an infant gut microbiota synthetic community in combinations of human milk oligosaccharides**

Athanasia Ioannou^1^, Maryse D. Berkhout^1^, William T. Scott Jr.^2,3^, Bernadet Blijenberg^4^, Sjef Boeren^5^, Marko Mank^4^, Jan Knol^4^, Clara Belzer^1*^

1. Laboratory of Microbiology, Wageningen University & Research, the Netherlands
2. Laboratory of Systems and Synthetic Biology, Wageningen University & Research, the Netherlands
3. UNLOCK, Wageningen University & Research and Delft University of Technology, Wageningen, the Netherlands
4. Danone Nutricia Research, Utrecht, the Netherlands
5. Laboratory of Biochemistry, Wageningen University & Research, the Netherlands

*Corresponding author email: clara.belzer@wur.nl

# Supplementary Materials & Methods

## Quality control of strains

The purity of in-house stocks was assessed with Sanger sequencing of the 16S rRNA gene after PCR amplification with the primers 27F and 1492R (Supplementary Table 2). The 16S rRNA gene copy number information per strain was retrieved from the following databases: rrnDB, NCBI, GTDB, IMG/JGI or the webpage of ATCC. Additionally, the ContEst16S tool of EZBioCloud (CJ Biosciences Inc., Seoul, Republic of Korea) was ran using the genome of the strain.

## Pre-cultures

All strains were pre-cultured from a glycerol stock solution in the basal medium. *Veillonella parvula* medium contained 14 ml/L Sodium DL-lactate solution (60% w/w). For *Lactobacillus rhamnosus* and *Ruminococcus gnavus* 60 mM D-glucose was used as carbon source. All other strains were grown in 20 mM lactose. Based on previous growth experiments, strains were grown at 37^o^C non-shaken for 24 h to 48 h to reach exponential phase. *Bacteroides* spp. were incubated for 48 h.

## Continuous fermentation quality control

Calibration of the inflow pumps, outflow pumps, pH probes and redox probes was performed prior to every experiment to ensure reproducibility. The medium was incubated overnight prior to inoculation to serve as a negative control. Growth was monitored with spectrophotometric measurements at OD600nm. The timepoint of transition to continuous fermentation was decided based on stable OD600nm measurements of the batch fermentation.

**qPCR standards preparation**

DNA was isolated using the FastDNA SPIN Kit for Soil (MP Biomedicals, California, USA) according to the manufacturer's protocol except for the homogenization step (3 times at 5.5 m/s for 60 sec instead of 6 m/s for 40 sec) and the additional incubation at 55^o^C for 5 min before the final centrifugation. The DNA yield was measured with the Qubit dsDNA BR Assay Kit. The strains used were: *Bifidobacterium infantis* ATCC15697, *Bifidobacterium bifidum* JCM1254, *Bifidobacterium breve* ATCC 15700, *Bacteroides fragilis* ATCC25285, *Bacteroides vulgatus* ATCC8482, *Bacteroides ovatus* ATCC8483 and *Escherichia coli* MG1655.

The standards were prepared by amplifying the 16S rRNA gene with primers 27F-1492R (Supplementary Table 2) in duplicate. The PCR mix was prepared with 10 μl of 5X Phusion HF Buffer (ThermoFisher Scientific, Massachusetts, USA), 1 μl dNTPs, 0.5 μl forward primer (10 μM), 0.5 μl reverse primer (10 μM), 0.5 μl Phusion Hot Start II High-Fidelity DNA Polymerase (ThermoFisher Scientific), 36.5 μl Nuclease Free water and 1 μl DNA template per well. The amplification program consisted of 1 cycle at 95^o^C for 5 min, 35 cycles of denaturation at 95^o^C for 30 sec, then annealing at 52^o^C for 20 sec and elongation at 72^o^C for 30 sec and a final extension step at 72^o^C for 7 min.

The PCR product was purified using the CleanNGS beads (CleanNA, Waddinxveen, the Netherlands) according to the manufacturer’s protocol. The concentration of the purified PCR products was measured with the Qubit dsDNA BR Assay Kit (ThermoFisher Scientific). Subsequently, the number of copies based on the concentration of each purified PCR product was calculated using the formula:

$$(Concentration(ng/\mu l)*10^23*6.022)/(10^9*1465bp*660)$$

Based on this, standards with a concentration from ${10}^{1}$to ${10}^{8}$ copies per μl were prepared in Nuclease Free water.

## Equations for HMO depletion calculation

$$Percent deviation from t0= \frac{Average technical replciates timepoint}{Average technical replicates t0} x 100$$

$$Percent degrdation=100-Percent deviation from t0$$

$$Relative standard deviation=\frac{Standard deviation of technical replicates}{Average of technical replciates} x 100$$

## Correction of 16S rRNA gene amplicon counts

16S rRNA gene copies per ml of culture:

$$16S copies= \frac{CN x DNA concentration x volume extract}{DNA concentration diluted}$$

CN: copy number of species or total bacteria as inferred by qPCR with primers for the specific species.

DNA concentration: DNA concentration of DNA extract from 1 ml of culture in ng/μl.

Volume extract: The volume of the DNA extract in μl.

DNA concentration diluted: DNA concentration of diluted DNA extract normalized for qPCR in ng/μl.

Relative abundance of species belonging to the *Bifidobacterium* and *Bacteroides* genus:

$$Species RA=\frac{Species i CN}{Sum CN species within genus} x Genus RA$$

Species i CN: copy number of species as inferred by qPCR with primers for the specific species.

Sum CN species within genus: the sum of copy numbers from all the species measured from each genus, as inferred by qPCR with primers for the specific species.

Genus RA: relative abundance of genus as inferred from the 16S rRNA gene amplicon analysis.

Cell number per ml of culture:

$$Cell numbers per ml= \frac{RA x total bacteria CN}{Species 16S CN}$$

RA: relative abundance of species as calculated from the 16S rRNA gene amplicon sequencing results or the equation above.

Total bacteria CN: copy number of total bacteria, as inferred by qPCR with primers targeting all bacterial species.

Species 16S CN: Species 16S rRNA gene copy number.

## Creation of genome-scale metabolic models

To perform the modeling analysis, genome-scale metabolic models (GEMs) were constructed using the CarveMe tool. The genome assemblies used in this study were acquired from the NCBI RefSeq database, ensuring a reliable and standardized source of genomic data. Specifically, the following genomes were used to create the GEMs (Supplementary Methods Table 1). Each genome assembly was downloaded and assessed for quality and to ensure high-quality data input for model construction. CarveMe was chosen for its ability to generate high-quality metabolic models from genome assemblies efficiently using a combination of automated genome annotation and gap-filling techniques to predict metabolic networks, resulting in comprehensive models. Default settings were used for the biomass equation of the model based on each species’ Gram status.

Supplementary Methods Table 1. Genomes used for the creation of genome-scale metabolic models.

| **Species** | **Strain** | **NCBI RefSeq Accession Number** |
| --- | --- | --- |
| *Bifidobacterium infantis* | ATCC15697 (JCM 1222/ DSM 20088) | GCF_000020425.1 |
| *Bifidobacterium bifidum* | JCM1254 (DSM 20082) | GCF_001311705.1 |
| *Bifidobacterium breve* | ATCC 15700 (DSM 20213/ JCM 1192) | GCF_000158015.1 |
| *Bacteroides fragilis* | ATCC25285 (VPI 2553 [EN-2; NCTC 9343]) | ATCC 25285* |
| *Bacteroides vulgatus* | ATCC8482 (DSM1447) | GCF_900624655.1 |
| *Bacteroides ovatus* | DSM1896 | GCF_001314995.1 |
| *Veillonella parvula* | ATCC10790 (DSM2008) | GCF_000024945.1 |
| *Ruminococcus gnavus* | ATCC 29149 | GCF_009831375.1 |
| *Escherichia coli* | MG1655 (DSM 18039) | GCF_000005845.2 |
| *Lactobacillus rhamnosus* | GG or HN001 (DR20) | GCF_000026505.1 |
| *Enterococcus faecalis* | ATCC19433 (DSM20478) | GCF_000392875.1 |
| *Streptococcus salivarius* | subps. *thermophilus* strain ATCC19258 (DSM20617) | GCF_010120595.1 |
| *Blautia producta* | JCM1471 (DSM2950) | GCF_000439125.1 |

*Note: The genome assembly for *Bacteroides fragilis* strain ATCC25285 was sourced from the ATCC (American Type Culture Collection) instead of NCBI RefSeq.

We curated the genome-scale models of the proven HMO-degrading species to include the HMO degradation potential previously reviewed by us and others [1, 2] and to include the HMO degradation pathways (Supplementary Methods Table 2).

Supplementary Methods Table 2. Curated reactions pertaining to HMO degradation.

| **Species** | **Reaction space** | **HMO** | **Reaction** | **Enzyme name** |
| --- | --- | --- | --- | --- |
| *B. infantis* | intracellular | 2’-FL / 3-FL | glc-gal-fuc_c-> lcts_c + fuc__L_c  gal-glc-fuc_c-> lcts_c + fuc__L_c | α-L-fucosidase |
|  |  | All HMOs | lcts_c -> glc__D_c + gal_c | β-1,4-galactosidase |
|  |  | 3’-SL / 6’-SL | glc-gal-neu5Ac_c -> lcts_c + acnam_c | 2,3-2,6-a-sialidase |
|  |  | LNT | glc-gal-glcNAc_c -> lcts_c + acgam_c | β-hexosaminidase/ β-1,6-N-acetylglucosaminidase |
|  |  |  | glc-gal-glcNAc-gal_c -> glc-gal-glcNAc_c + gal_c | β-galactosidase |
| *B. bifidum* | extracellular | 2’-FL / 3-FL | glc-gal-fuc_c -> lcts_c + fuc__L_c  gal-glc-fuc_c-> lcts_c + fuc__L_c | α-1,3/1,4-L-fucosidase  α-1,2-L-fucosidase |
|  |  | 3’-SL / 6’-SL | glc-gal- neu5Ac_c -> lcts_c + acnam_c | 2,3-2,6-a-sialidase |
|  |  | LNT | glc-gal-glcNAc-gal_c -> glc-gal-glcNAc_c + gal_c  glc-gal-glcNAc_c -> lcts_c + acgam_c  glc-gal-glcNAc-gal_c -> lcts_c + glcNAc-gal | β-galactosidase  β-hexosaminidase/ β-1,6-N-acetylglucosaminidase  lacto-N-biosidase |
|  |  | LNB | gal-glcNAc_c -> gal_c + acgam_c | GNB/LNB phosphorylase |
|  |  | All HMOs | lcts_c -> glc__D_c + gal_c | β-1,4-galactosidase |
|  |  |  |  |  |
|  |  |  |  |  |
|  |  |  |  |  |
| *B. breve* | intracellular | 2’-FL / 3-FL | No utilization |  |
|  |  | 3’-SL / 6’-SL | No utilization |  |
|  |  | LNT | glc-gal-glcNAc-gal_c -> glc-gal-glcNAc_c+ gal_c  glc-gal-glcNAc_c -> lcts_c + acgam_c | β-galactosidase  β-hexosaminidase/ β-1,6-N-acetylglucosaminidase |
|  |  |  | gal-glcNAc_c -> gal_c + acgam_c | GNB/LNB phosphorylase |
|  |  | All HMOs | lcts_c -> Glc__D_c + gal_c | β-1,4-galactosidase |
|  |  |  |  |  |
|  |  |  |  |  |
|  |  |  |  |  |
| *B. fragilis* | Putatively extracellular* | 3’-SL / 6’-SL | glc-gal- acnam_e -> lcts_e + acnam_e | ɑ2-6/ɑ2-3 sialidase |
|  |  |  |  |  |
|  |  |  |  |  |
|  |  | 2’-FL / 3-FL | & | Fucosidase  Putative action on hmos |
|  |  |  |  |  |
|  |  | LNT | & | Acetylglucosaminidase |
|  |  | All HMOs | lcts_c -> glc__D_c + gal_c | β-galactosidase |
| *B. vulgatus* | Putatively extracellular* | 3’-SL / 6’-SL | glc-gal-fuc_e -> lcts_e + acnam_e | ɑ2-6/ɑ2-3 sialidase |
|  |  | 2’-FL / 3’-FL | glc-gal-fuc_e -> lcts_e + fuc__L_e | fucosidase |
| *R. gnavus* | Putatively extracellular** | 2’-FL / 3’-FL | glc-gal-fuc_e -> lcts_e + fuc__L_e | fucosidase |

& No reaction was curated into the genome-scale model for these conditions/HMOs because there is no single Gene-Protein-Reaction association we can ascribe to these related enzymes.

In this study, we applied constraints to a metabolic model using parsimonious flux balance analysis (pFBA) to simulate conditions that reflect a minimal medium composition under anaerobic conditions, specifically in the presence of both lactose and glucose. To model anaerobic conditions, oxygen uptake was entirely restricted by setting the bounds of the oxygen exchange reaction to zero (EX_o2_e: 0, 0). This constraint ensures that the model operates under strictly anaerobic conditions. The uptake of glucose and lactose was constrained to a lower bound of -10, allowing these sugars to be available to the model in controlled quantities. This was implemented by setting the bounds of the glucose (EX_glc__D_e) and lactose (EX_lcts_e) uptake reactions to -10 and 0, respectively. Additionally, constraints were applied to several human milk oligosaccharides (HMOs) and related substrates, including fucosyllactose and lacto-N-tetraose, by setting their uptake bounds to -5 and 0. A custom-defined medium was also incorporated into the model to include essential nutrients and HMOs, with specific uptake limits tailored to each component. For example, the uptake of glucose and lactose was allowed up to 10 units, while several other compounds, such as calcium (EX_ca2_e), iron (EX_fe2_e), and thiamine (EX_thm_e), were included with higher or lower uptake capacities depending on their physiological relevance. Finally, the model was configured to use the GLPK solver, and a comprehensive summary of the model was generated to verify the accuracy of the applied constraints and medium composition.

## Critical evaluation of GEMs

### **Incorporation of HMO degradation in GEMs**

Modelling HMO utilization presents inherent challenges related to incomplete annotations and a limited understanding of metabolic pathways. HMO degradation is not adequately characterized for some species, especially those in the *Bacteroides* genus. For example, we did not include HMO degradation pathways for *B. fragilis* due to uncertainties in the enzymatic sequences involved. Additionally, gaps exist in our understanding of the regulation of degrading enzymes. While the NagR regulator has been characterized in bifidobacteria [3], it remains unclear how other HMO components regulate enzyme expression in different species. Moreover, the transportation of HMOs is more complex than often described. Some HMOs, like 2'-FL, have characterized transporters facilitating their import into the cell. However, transporters for other HMOs, such as sialylated ones, have not been described in detail [2]. Transporters may have varying affinities for different HMOs, and general transporters may also play a role [4]. Additionally, HMO subcomponents might be exported to relieve osmotic pressure, as suggested previously [5]. These complexities hinder the accurate representation of HMO degradation in GEMs and likely contribute to discrepancies between model predictions and experimental observations. In our models, we attempted to incorporate HMO degradation pathways where possible, but the limitations mentioned above mean that the models may not fully capture the metabolic capabilities related to HMO utilization.

### **Differences between *in silico* and *in vitro* metabolite production**

Comparing the metabolite production of BIG-Syc members *in vitro* with the predictions from the GEMs (Supplementary Methods Table 3) revealed several discrepancies.

Supplementary Methods Table 3. Per-species metabolite production *in vitro* vs. *in silico* predictions.

| Species | Metabolites produced *in vitro* | Metabolites predicted by GEMs |
| --- | --- | --- |
| *B. infantis* | Acetate, formate, succinate, lactate, ethanol, CO_2_ | Succinate, acetaldehyde, 2 methylpropanoic acid, 4-Hydroxy-benzyl alcohol, CO_2_ |
| *B. bifidum* | Acetate, formate, succinate, lactate, ethanol, CO_2_ | 4-Hydroxy-benzyl alcohol, acetaldehyde, succinate, CO_2_, hydrogen sulfide |
| *B. breve* | Acetate, formate, lactate, CO_2_ | Acetaldehyde, succinate, CO_2_, oxalate, hydrogen sulfide |
| *B. fragilis* | Acetate, propionate, formate, succinate, lactate, 1,2-PDO, CO_2_ | Acetate, acetoacetate, acetaldehyde, CO_2_, glycolaldehyde, hydrogen sulfide |
| *B. vulgatus* | Acetate, propionate, succinate, CO_2_ | Isobutyrate, ethanol, propanoyl phosphate, CO_2_ |
| *B. ovatus* | Formate, CO_2_ | 4-Hydroxy-benzyl alcohol, glycolaldehyde, hydrogen sulfide, isobutyrate, succinate, CO_2_ |
| *E. coli* | Acetate, formate, succinate, lactate, ethanol, 1,2-PDO, CO_2_, H_2_ | 4-Hydroxy-benzyl alcohol, ethanol, hydrogen sulfide, formate, succinate, CO_2_ |
| *B. producta* | Acetate, formate, lactate, ethanol, 1,2-PDO, CO_2_, H_2_ | 1,3-Propanediol, acetaldehyde, hydrogen sulfide, propanoyl phosphate, CO_2_ |
| *E. faecalis* | Acetate, formate, lactate, ethanol, 1,2-PDO, CO_2_ | 2 methylpropanoate, acetaldehyde, glycolaldehyde, H_2_, hydrogen sulfide, CO_2_ |
| *L. rhamnosus* | Formate, ethanol | Acetaldehyde, hydrogen sulfide, 2 methylpropanoate, glycolaldehyde, propanoyl phosphate, CO_2_ |
| *R. gnavus* | Acetate, propionate, formate, succinate, 1,2-PDO, 1-propanol, ethanol, CO_2_ | Citrate, acetaldehyde, 4-Hydroxy-benzyl alcohol, hydrogen sulfide, 4-methylphenol, CO_2_ |
| *V. parvula* | Acetate, formate, succinate, propionate, CO_2_, H_2_ | Acetaldehyde, glycolaldehyde, hydrogen sulfide, propanoyl phosphate, CO_2_ |
| *S. salivarius subsp. thermophilus* | - | 2 methyl butanoate, 2 methylpropanoate, 3 methylbutanoate, acetaldehyde, hydrogen sulfide, glycolaldehyde, CO_2_ |

Possible reasons for these differences include:

**Growth Rates and Maintenance Energy**: Some species, such as *B. ovatus* and *L. rhamnosus*, exhibited minimal growth *in vitro*, as evidenced by low biomass accumulation and limited metabolite production. For instance, *B. ovatus* primarily produced formate and CO₂ *in vitro*, while the GEM predicted additional metabolites like hydrogen sulfide and succinate. Similarly, *L. rhamnosus* showed minimal metabolite production *in vitro,* producing only formate and ethanol, whereas the GEM predicted multiple metabolites not observed experimentally. These observations suggest that under the experimental conditions, these organisms may allocate more energy toward cellular maintenance rather than growth, leading to reduced metabolic outputs. The GEMs may not accurately represent these energy allocation strategies because they often assume optimal growth conditions without accounting for increased maintenance energy requirements under stress or nutrient limitations. As a result, the models may overpredict metabolite production or predict metabolites that are not produced in significant amounts *in vitro*.

**Stress Responses**: Under low carbon source stress, bifidobacteria are known to shift their metabolic pathways, producing more acetate, ethanol, and formate, and less lactate [6]. In our *in vitro* experiments, these species produced significant amounts of acetate and formate, and lactate to a lesser extent. For example, *B. infantis* produced acetate, formate, and ethanol *in vitro*, while the GEMs predicted metabolites like acetaldehyde and 2-methylpropanoic acid instead of ethanol. Such stress-induced metabolic shifts may not be captured in the GEMs, which assume optimal growth conditions with ample carbon sources and lack regulatory mechanisms that adjust metabolic fluxes in response to environmental stresses, leading to discrepancies between predicted and observed metabolite profiles.

To accurately model stress responses, genome-scale metabolic models need to incorporate dynamic and regulatory features. Approaches like dynamic flux balance analysis (dFBA) and regulatory FBA (rFBA) allow the simulation of time-dependent changes and regulatory adjustments in metabolic fluxes under stress conditions. However, implementing these methods requires extensive data on gene regulation, enzyme kinetics, and environmental dynamics—such as time-resolved gene expression profiles and kinetic parameters under stress—which were not available for our study organisms. Due to these data limitations and the increased computational complexity, we did not employ dFBA or rFBA in this study. Consequently, our models do not reflect the adaptive metabolic shifts that occur under low carbon availability, limiting their predictive accuracy under stress conditions.

**Technical Factors in Metabolite Production**: Factors such as pH changes during *in vitro* experiments can significantly affect bacterial growth and metabolism. For instance, the accumulation of organic acids like acetate and lactate can lower the pH, inhibiting the growth of pH-sensitive species such as *E. coli* and *B. fragilis* [7]. In our experiments, the pH was controlled to 6 with base addition. However, BIG-Syc members produced multiple acids, which may have led to self-inhibition due to topical pH reduction.

Our GEMs do not account for pH fluctuations, assuming a constant optimal pH environment. Incorporating pH effects into GEMs would require detailed thermodynamic data adjusted for pH, including standard Gibbs free energy changes (ΔG⁰') for reactions at different pH levels. It would also necessitate enzyme kinetics information, such as pH-dependent kinetic parameters (Km and Vmax), and knowledge of metabolite protonation states and their speciation under varying pH conditions. Additionally, proton balancing in reactions and modelling of proton gradients across membranes would be essential.

Due to the complexity and lack of comprehensive pH-dependent data for all enzymes and metabolites in the network, our models currently lack these features. This limitation could lead to discrepancies where the GEMs predict the production of metabolites that are not observed *in vitro* due to pH-induced metabolic inhibition. For example, the models may overestimate the growth and metabolic activity of *E. coli* under acidic conditions that, in reality, inhibit its metabolism.

Furthermore, the buffering capacity of the medium and the rate of acid production can impact the microbial community dynamics, affecting factors like nutrient availability and interspecies interactions, which are not captured in the individual GEMs. Including pH effects in the models would improve their physiological relevance and predictive accuracy but would require significant additional data collection and model refinement, including integration of proteomic data to account for enzyme abundances and activities under different pH conditions

**Modeling Tools and Assumptions**: We used CobraPy for FBA of our GEMs. CobraPy employing FBA operates under the assumption of steady-state conditions, meaning that metabolite concentrations remain constant over time, and it does not account for dynamic changes in the environment or cellular regulation. This assumption may not reflect the actual biological conditions in our *in vitro* experiments. For example, during the fermentation process, the accumulation of organic acids like acetate and lactate can lead to feedback inhibition of metabolic pathways or alter enzyme activities, but these effects are not captured in the steady-state FBA models. In the case of *E. coli*, the accumulation of acetate might inhibit its growth and metabolism, leading to discrepancies between predicted and observed metabolite production.

Additionally, CobraPy-based FBA does not incorporate regulatory effects or kinetic limitations. It assumes that all enzymes operate at maximum capacity and that gene expression remains constant, which may not be true under experimental conditions. For instance, bacteria may downregulate certain metabolic pathways in response to nutrient availability or stress, resulting in decreased production of certain metabolites. Since the FBA models do not account for gene regulatory mechanisms or enzyme kinetics, they may overpredict production compared to what is observed experimentally. These assumptions can impact the accuracy of predictions by not reflecting the actual metabolic adjustments organisms make in response to their environment.

**Substrate Availability and Medium Composition**: In our *in vitro* experiments, the medium composition and substrate availability differed from the assumptions made in the GEMs. The models typically assume constant and ample availability of carbon sources like lactose and HMOs, without accounting for depletion over time or competition between species. However, in our synthetic HMO-degrading microbial community, substrates are consumed at varying rates by different organisms. For example, *B. infantis* is highly efficient at consuming specific HMOs such as 2'-FL. If *B. infantis* rapidly depletes 2'-FL from the medium, other species that also utilize this substrate may experience limited availability, affecting their growth and metabolism. The GEMs do not capture this dynamic depletion and assume that all species have equal and constant access to all substrates, leading to discrepancies in predicted metabolite production.

Moreover, the models do not account for species interactions like competition and cross-feeding that occur in the microbial community. In our *in vitro* system, metabolites produced by one species can serve as substrates for another. For instance, the lactate produced by *B. infantis*. Since individual GEMs cannot model these interspecies dynamics and substrate competition, they may inaccurately predict metabolite profiles compared to the *in vitro* observations. This limitation highlights the need for community-level modelling approaches that incorporate substrate availability changes and microbial interactions to improve predictive accuracy.

**Community Interactions**: It is important to note that microbial community modelling approaches, such as community flux balance analysis (cFBA) [8], and tools like SteadyCom [9], and MICOM [10], were not applied using the GEMs created in this study. This represents a limitation, as these methods are capable of simulating interactions within microbial communities and have been identified as viable options for modelling analysis [11]. Our focus was on individual GEMs, and thus, the potential cross-feeding or competitive interactions between species were not explored. In our *in vitro* experiments, we cultured the synthetic HMO-degrading microbial community (BIG-Syc) as a co-culture, where multiple microbial species interact within the same environment. These interactions can significantly influence metabolite production, but our GEMs simulate each species individually, without accounting for such interactions. For example, cross-feeding is a common phenomenon where one species consumes metabolites produced by another. *B. infantis* ferments HMOs to produce acetate and lactate, and the latter can be utilized by *V. parvula*. Another example is the competitive interaction for substrate utilization among community members. Both *B. infantis* and *B. breve* can utilize certain HMOs like LNT. In the co-culture, these species may compete for LNT, affecting their growth rates and metabolic outputs. If *B. infantis* outcompetes *B. breve* for LNT, *B. breve* may shift its metabolism to utilize alternative substrates or produce different metabolites. These competitive dynamics can lead to changes in metabolite profiles that are not captured when modelling species individually. The GEMs assume constant substrate availability and do not account for the depletion of HMOs due to competition, leading to discrepancies between predicted and observed metabolite production in the co-culture. These examples highlight the complexity of microbial interactions and the limitations of individual GEMs in predicting community-level metabolic outcomes.

The aforementioned factors highlight the limitations of our GEMs in accurately predicting metabolite production and underscore the need for more comprehensive models that incorporate environmental conditions, regulatory mechanisms, and microbial interactions.

If one wanted to utilize GEMs for modelling *in vivo* microbial interactions, one would need to adapt the models further based on the aforementioned characteristics of the gut environment. In the human luminal environment, nutrients are spatiotemporally distributed because feeding occurs at certain intervals. Food components and metabolites produced by the gut microbiota may not be perfectly mixed and may degrade at different rates upon reaching the gut. Moreover, feces are in proximity to the mucosal layer, which constantly sheds mucin, creating a gradient that influences microbial activity. Efforts to simulate these gradients have been addressed elsewhere [12]. Additionally, the lumen exhibits one of the steepest oxygen gradients in the human body [13]. Other gases may also not be uniformly available, especially since stool hardness can impede their penetration. All these factors did not apply to our *in vitro* fermentation system, where there is continuous mixing, sparging of gases, maintenance of pH and redox potential, and continuous addition of nutrients. Here, we provided a homogeneous environment that does not replicate the gradients and spatial heterogeneity present in the gut. This could involve integrating spatial gradients, variable nutrient availability, regulatory mechanisms, and interactions with host tissues to more accurately reflect *in vivo* conditions. Incorporating such complexities into GEMs would enhance their predictive accuracy and biological relevance, addressing the limitations highlighted in our study.

### **Relevance to our study**

Despite the discrepancies between the GEM predictions and the *in vitro* metabolite profiles of individual species in our synthetic HMO degrading microbial community, the GEMs remain valid and useful for our study's purposes. Firstly, the GEMs provide valuable qualitative insights into the metabolic pathways and capabilities of the community members, supporting our proteomics data. For example, while the GEM for *B. infantis* did not quantitatively predict the exact production of acetate and formate as observed *in vitro*, it correctly included the pathways for HMO degradation and the potential to produce key metabolites like succinate and CO₂. This aligns with recent proteomic studies showing the expression of enzymes involved in HMO utilization and central metabolism in *B. infantis* [14]. Similarly, the GEMs for *B. bifidum* and *B. breve* captured essential aspects of their metabolism, such as succinate production and the presence of lactate dehydrogenase, which is supported by recent findings indicating active fermentation pathways in these species when grown on HMOs [15]. This integration enhances our understanding of each species' metabolic role within the community, even if the specific metabolite quantities differ from experimental results.

Secondly, the GEMs are effective in predicting the production of gases, which is a critical aspect of our study. For instance, the models successfully predicted CO₂ production across multiple species, including *B. infantis*, *B. bifidum*, *E.coli*, and *B. fragilis*, aligning with *in vitro* observations. Gas production often results from central metabolic pathways that are well-conserved and accurately represented in GEMs. Even though the GEM for *E. coli* did not predict all soluble metabolites correctly, it did simulate ethanol and CO₂ production, which are significant fermentation products under anaerobic conditions [16]. This makes GEMs valuable for predicting gas fluxes within the community, providing insights into fermentation processes and the overall metabolic dynamics. Recent studies have shown that GEMs can reliably predict gas production in microbial communities, even when quantitative predictions of other metabolites are less accurate [17–19]. Therefore, despite quantitative inaccuracies in certain metabolite predictions, the GEMs remain useful tools for supporting proteomics data and understanding gas production in our synthetic microbial community.

# Supplementary information

Supplementary Table 1. HMO structural characteristics of the 4HMO and the 5HMO mix (concentration 4 gr/L) and human milk data derived from a previous study [20].

| **Criteria** | **5HMO mix (μmol/ml)** | **4HMO mix (μmol/ml)** | **Min human milk (μmol/ml)** | **Max human milk (μmol/ml)** |
| --- | --- | --- | --- | --- |
| HMO-bound sialic acid | 0.612 | 2.29816 | 1.51 | 3.84 |
| HMO-bound fucose | 4.91796 | 4.8972 | 6.618 | 14.835 |
| Type 1 | 1.46976 | 0 | 3.25 | 6.689 |
| Type 2 | 0 | 0 | 0.604 | 1.746 |
| alpha 1-2 | 3.968 | 3.0628 | 2.345 | 9.316 |
| alpha 1-3 | 0.94996 | 1.8344 | 0.127 | 0.789 |
| alpha 2-6 | 0.328 | 1.71732 | 0.399 | 1.361 |
| Total HMO | 6.99972 | 7.19536 | 10.045 (secretors)  7.048 (non-secretors) | 18.093 (secretors)  14.354 (non-secretors) |
| alpha 1-4 present | no | no | yes | |
| alpha 2-3 present | yes | yes | yes | |

Supplementary Table 2. Primers used in this study.

| **Species** | **Primer** | **Citation** | **T_a_** | **Amplicon** |
| --- | --- | --- | --- | --- |
| V5-V6 region of the 16S rRNA gene | 784F: 5'-RGGATTAGATACCC-3’  1064R: 5'-CGACRRCCATGCANCACCT-3’ |  | 42^o^C | 280 bp |
| 16S rRNA gene complete | 27F:5'-AGAGTTTGATCMTGGCTCAG-3’  1492R:5'–TACGGYTACCTTGTTACGACTT-3’ |  | 52^o^C | 1465 bp |
| Total bacteria | 1048F:5`-GTGSTGCAYGGYYGTCGTCA-3`  1175R:5`-ACGTCRTCCMCNCCTTCCTC-3’ | [21] | 52^o^C | 127 bp |
| *B. infantis* | BiINF-1:5`-TTCCAGTTGATCGCATGGTC-3’  BiINF-2:5`-GGAAACCCCATCTCTGGGAT-3’ | [22] | 55^o^C | 828 bp |
| *B. bifidum* | BiBIF-1:5`-CCACATGATCGCATGTGATTG-3’  BiBIF-2:5`-CCGAAGGCTTGCTCCCAAA-3’ | [22] | 55^o^C | 278 bp |
| *B. breve* | BiBRE-1:5`-CCGGATGCTCCATCACAC-3’  BiBRE-2:5`-ACAAAGTGCCTTGCTCCCT-3’ | [22] | 55^o^C | 288 bp |
| *B. fragilis* | s-Bfra186-F:5`-AATGATTCCGCATGGTTTCA-3’  s-Bfra592-R:5`-CAAACTTTCACAACTGACTTAC-3 | [22] | 55^o^C | 425 bp |
| *B. vulgatus* | s-Bvul129-F:5`-AACCTGCCGTCTACTCTT-3’  s-Bvul585-R:5`-CAACTGACTTAAACATCCAT-3’ | [22] | 55^o^C | 473 bp |
| *B. ovatus* | s-Bova175-F:5`-CCGGATAGCATACGAAYAT  s-Bova587-R:5`-CACAACTGACTTAACAATCC | [22] | 55^o^C | 428 bp |

Supplementary Table 3. Representative amino acid sequences used as queries in BLASTp search.

| **UniProt identifier** | **EC number (GH family)** | **Enzyme type** | **Host microorganism** |
| --- | --- | --- | --- |
| E8MF13 · LNPA_BIFL2 | EC:2.4.1.211 (GH112) | **1,3-beta-galactosyl-N-acetylhexosamine phosphorylase** | *Bifidobacterium longum* subsp. longum ATCC 15707 |
| B7GNQ0 · B7GNQ0_BIFLS | EC:3.2.1.18 (GH33) | **Exo-alpha-sialidase** | *Bifidobacterium longum* subsp. infantis ATCC 15697 |
| A0A829NK98 · A0A829NK98_RUMGN | EC:3.2.1.18 (GH33) | **Anhydrosialidase** | *[Ruminococcus] gnavus* CC55_001C |
| B7GNN6 · B7GNN6_BIFLS | EC: 3.2.1.23 (GH2) | Beta-galactosidase | *Bifidobacterium longum* subsp. infantis ATCC 15697 |
| B7GUD7 · B7GUD7_BIFLS | EC: 3.2.1.23 (GH42) | Beta-galactosidase | *Bifidobacterium longum* subsp. infantis ATCC 15697 |
| H6WX44 · H6WX44_9GAMM | EC: 3.2.1.23 (GH1) | Beta-galactosidase BgalH | *Halomonas spp.* |
| B2UQ71 · B2UQ71_AKKM8 | EC: 3.2.1.23 (GH35) | Beta-galactosidase | *Akkermansia muciniphila* ATCC BAA-835 |
| B7GNN8\|B7GNN8_BIFLS | EC: 3.2.1.51 (GH29) | Alpha-1,3/4-fucosidase | *Bifidobacterium longum* subsp. infantis ATCC 15697 |
| B7GN69\|B7GN69_BIFLS | EC: 3.2.1.52 (GH20) | Beta-N-acetylhexosaminidase | *Bifidobacterium longum* subsp. infantis ATCC 15697 |
| B7GNN7\|B7GNN7_BIFLS | EC: 3.2.1.63 (GH95) | Glyco_hyd_65N_2 domain-containing protein | *Bifidobacterium longum* subsp. infantis ATCC 15697 |
| A0A454XTE7\|A0A454XTE7_LACCD | EC: 3.2.1.85 (GH1) | 6-phospho-beta-galactosidase | *Lactobacillus casei* BD-II |
| B7GPC7\|EBI1_BIFLS | EC: 3.2.1.96 (GH18) | Endo-beta-N-acetylglucosaminidase | *Bifidobacterium longum* subsp. infantis ATCC 15697 |
| B3DQP7\|B3DQP7_BIFLD | EC: 3.2.1.96 (GH85) | Mannosyl-glycoprotein endo-beta-N-acetylglucosaminidase | *Bifidobacterium longum* DJO10A |
| A0A6B8LQN9\|A0A6B8LQN9_9BIFI | EC: 3.2.1.111 (GH29) | Alpha-1 3/4-fucosidase | *Bifidobacterium catenulatum* subsp. kashiwanohense |
| B3TLD6\|B3TLD6_BIFBI | EC: 3.2.1.140 (GH20) | Beta-N-acetylhexosaminidase | *Bifidobacterium bifidum* JCM 1254 |
| A0A024QYS6\|A0A024QYS6_BIFLN | EC: 3.2.1.140 (GH136) | Lacto-N-biosidase | *Bifidobacterium longum* subsp. longum |

Supplementary Table 4. UniProt metrics on amino acid sequence retrieval from EIBER protein groups.

| **UniProt metrics on retrieval of amino acid sequences of protein groups in the 3 term infants of the EIBER study** | **UniProt metrics on retrieval of amino acid sequences of protein groups in the 1 vaginally born term infant of the EIBER study** |
| --- | --- |
| **1,087** IDs were mapped to **2,379** results  **205**ID were not mapped:   - D5C9X9_ENTCC - D5CDZ0_ENTCC - I3U1N6_ENTFC - Q6A7S9_PROAC - D5CGW3_ENTCC - RL5_PROAC - D5CDI1_ENTCC - D5CEL4_ENTCC - Q3XWW9_ENTFC - D5CGE8_ENTCC - D5C6R3_ENTCC - D5CDZ4_ENTCC - I3TYN0_ENTFC - T1ZYM9_STRAP - G7UXM6_LACRH - Q3XWH5_ENTFC - D5CBP6_ENTCC - Q6A9J4_PROAC - W6G2D1_LACPA - A0A023VFQ1_CITFR - T2A1W0_STRAP - Q6A690_PROAC - T2A3J2_STRAP - Q6ABH4_PROAC - D5C6N5_ENTCC - Q3Y2I1_ENTFC - Q3XWN8_ENTFC - D5CBQ2_ENTCC - W6GFE8_LACPA - D5CE97_ENTCC - Q3XZ32_ENTFC - D5C7P7_ENTCC - Q3XYX0_ENTFC - Q8G848_BIFLO - V5VCC7_ACIBA - D5C732_ENTCC - I3TY42_ENTFC - D5C6W9_ENTCC - RL19_PROAC - Q3Y184_ENTFC - I3U1N4_ENTFC - D5CDF6_ENTCC - CH602_PROAC - D5CC12_ENTCC - Q18C45_PEPD6 - W6G283_LACPA - D5CI71_ENTCC - H6P935_STRIC - A0A023V969_CITFR - D5C6R8_ENTCC - Q3XYW2_ENTFC - A0A023V9K1_CITFR - D4L9G8_9FIRM - A0A023V7X6_CITFR - V5VA58_ACIBA - D5CF84_ENTCC - D5C7N1_ENTCC - A0A023V4D9_CITFR - D5CDF1_ENTCC - D5CHC8_ENTCC - E3GQ72_EUBLK - D5C7R1_ENTCC - G3P3_ECOLI - A0A023V3F4_CITFR - Q6A7Q8_PROAC - Q5AKX0_CANAL - T1ZZF9_STRAP - I3U3V3_ENTFC - H6P8S7_STRIC - D5C8L5_ENTCC - R6QZ37_9FIRM - D5CHU6_ENTCC - A0A023V4D0_CITFR - Q3XZA0_ENTFC - T2A292_STRAP - Q3Y2B1_ENTFC - H6P9K5_STRIC - H6P9Y3_STRIC - A0A023V2V4_CITFR - A0A023VDK2_CITFR - D5CB12_ENTCC - Q6A9W6_PROAC - RS2_PROAC - A0A023V726_CITFR - D5CBU1_ENTCC - D5CBR3_ENTCC - W6G4E4_LACPA - A0A023VEY7_CITFR - D5C6R7_ENTCC - RS4_PROAC - T1ZXR8_STRAP - D5CHT8_ENTCC - D5CDZ3_ENTCC - D5CDI0_ENTCC - A0A023V6S7_CITFR - H6P9V2_STRIC - D5C7T3_ENTCC - Q3XXW8_ENTFC - Q3Y121_ENTFC - RS17_PROAC - D5C9Q8_ENTCC - D5CIV5_ENTCC - T2A3W5_STRAP - T2A2A0_STRAP - D5CI01_ENTCC - R6QY92_9FIRM - R6Y665_9PORP - Q3Y200_ENTFC - R6XC86_9PORP - D5CJZ6_ENTCC - D5CDH4_ENTCC - D5CD89_ENTCC - A0A023V8I8_CITFR - I3TYM6_ENTFC - I3TYB5_ENTFC - D5CDG7_ENTCC - D5CHT4_ENTCC - D5CC60_ENTCC - D5CE41_ENTCC - D5CIU2_ENTCC - D5CHU2_ENTCC - D5CGQ2_ENTCC - A0A023V3S4_CITFR - R6Y0V0_9PORP - D5CH37_ENTCC - D5CGY4_ENTCC - D5CCW4_ENTCC - I3TY41_ENTFC - T2A3A5_STRAP - PDXS_PROAC - T2A2Z5_STRAP - I3U058_ENTFC - D5CC19_ENTCC - Q6A9N2_PROAC - I3U5I6_ENTFC - A0A023V821_CITFR - A0A023V3F0_CITFR - Q1WTB3_LACS1 - H6P8R8_STRIC - D5CDF7_ENTCC - I3U2S9_ENTFC - D5C871_ENTCC - D5C6F5_ENTCC - G7UZE4_LACRH - H6P9A7_STRIC - ENO_PROAC - A0A023VD31_CITFR - GTPC1_CLOPE - A0A023V983_CITFR - D5CDG9_ENTCC - T2A2Q7_STRAP - Q3Y285_ENTFC - D5CFQ9_ENTCC - T1ZZ00_STRAP - H6PCK0_STRIC - D5CEU5_ENTCC - A0A023V325_CITFR - D5CED2_ENTCC - D5CGF1_ENTCC - Q3XYX3_ENTFC - RPOC_PROAC - RL2_PROAC - T2A3D5_STRAP - H6PAQ8_STRIC - D5CC00_ENTCC - V5VFT2_ACIBA - D5CDF0_ENTCC - A0A023NW04_9GAMM - G2SMP0_LACRR - T2A0N7_STRAP - G2SPN2_LACRR - H6PCJ8_STRIC - D5C883_ENTCC - A0A023VF65_CITFR - H6PAQ5_STRIC - G2SR82_LACRR - Q3XYX5_ENTFC - Q6A6D1_PROAC - Q3Y1L8_ENTFC - H6PCL1_STRIC - D5CF02_ENTCC - Q3Y122_ENTFC - A0A023V7D7_CITFR - W6GFN3_LACPA - D5CCK4_ENTCC - D5CDF8_ENTCC - D5CDH3_ENTCC - RPOB_PROAC - D5CF33_ENTCC - Q838S2_ENTFA - D5C730_ENTCC - EFTU_PROAC - T2A1Q3_STRAP - A0A023V3X2_CITFR - D5C728_ENTCC - A0A023V7E0_CITFR - Q3XX08_ENTFC - D5CH16_ENTCC - R6XBD7_9PORP - T1ZZY5_STRAP - RS7_PROAC - H6PCJ5_STRIC - D5CF78_ENTCC - H6PCS3_STRIC - A0A095MA10_BURML - Hide IDs   **946**[active](https://www.uniprot.org/id-mapping/uniprotkb/3aec890e44d52592222bbbdff5d4594049e88ac7/overview?query=active%3Atrue) entries and**141** [obsolete](https://www.uniprot.org/id-mapping/uniprotkb/3aec890e44d52592222bbbdff5d4594049e88ac7/overview?query=active%3Afalse) entries are found | **883**IDs were mapped to **1,931** results  **165**ID were not mapped:   - D5C9X9_ENTCC - D5CDZ0_ENTCC - I3U1N6_ENTFC - D5CGW3_ENTCC - D5CDI1_ENTCC - D5CEL4_ENTCC - Q3XWW9_ENTFC - D5C6R3_ENTCC - D5CDZ4_ENTCC - I3TYN0_ENTFC - G7UXM6_LACRH - Q3XWH5_ENTFC - D5CBP6_ENTCC - Q6A9J4_PROAC - W6G2D1_LACPA - A0A023VFQ1_CITFR - T2A1W0_STRAP - Q6A690_PROAC - T2A3J2_STRAP - Q3Y2I1_ENTFC - D5CBQ2_ENTCC - W6GFE8_LACPA - D5CE97_ENTCC - Q3XZ32_ENTFC - D5C7P7_ENTCC - Q3XYX0_ENTFC - Q8G848_BIFLO - V5VCC7_ACIBA - D5C732_ENTCC - I3TY42_ENTFC - D5C6W9_ENTCC - RL19_PROAC - Q3Y184_ENTFC - D5CDF6_ENTCC - CH602_PROAC - Q18C45_PEPD6 - D5CI71_ENTCC - H6P935_STRIC - A0A023V969_CITFR - D5C6R8_ENTCC - A0A023V9K1_CITFR - A0A023V7X6_CITFR - V5VA58_ACIBA - D5CF84_ENTCC - D5C7N1_ENTCC - A0A023V4D9_CITFR - D5CDF1_ENTCC - D5CHC8_ENTCC - E3GQ72_EUBLK - D5C7R1_ENTCC - G3P3_ECOLI - A0A023V3F4_CITFR - Q5AKX0_CANAL - T1ZZF9_STRAP - I3U3V3_ENTFC - H6P8S7_STRIC - R6QZ37_9FIRM - D5CHU6_ENTCC - A0A023V4D0_CITFR - Q3XZA0_ENTFC - T2A292_STRAP - H6P9K5_STRIC - H6P9Y3_STRIC - A0A023V2V4_CITFR - D5CB12_ENTCC - Q6A9W6_PROAC - RS2_PROAC - A0A023V726_CITFR - D5CBU1_ENTCC - RS4_PROAC - T1ZXR8_STRAP - D5CHT8_ENTCC - D5CDZ3_ENTCC - D5CDI0_ENTCC - A0A023V6S7_CITFR - H6P9V2_STRIC - D5C7T3_ENTCC - Q3Y121_ENTFC - RS17_PROAC - D5C9Q8_ENTCC - D5CIV5_ENTCC - T2A3W5_STRAP - T2A2A0_STRAP - D5CI01_ENTCC - R6QY92_9FIRM - R6Y665_9PORP - Q3Y200_ENTFC - R6XC86_9PORP - D5CJZ6_ENTCC - D5CDH4_ENTCC - D5CD89_ENTCC - A0A023V8I8_CITFR - I3TYB5_ENTFC - D5CDG7_ENTCC - D5CHT4_ENTCC - D5CC60_ENTCC - D5CE41_ENTCC - D5CHU2_ENTCC - D5CGQ2_ENTCC - A0A023V3S4_CITFR - D5CGY4_ENTCC - D5CCW4_ENTCC - I3TY41_ENTFC - PDXS_PROAC - T2A3A5_STRAP - D5CC19_ENTCC - Q6A9N2_PROAC - I3U5I6_ENTFC - A0A023V821_CITFR - A0A023V3F0_CITFR - Q1WTB3_LACS1 - D5CDF7_ENTCC - D5C871_ENTCC - H6P9A7_STRIC - ENO_PROAC - A0A023VD31_CITFR - GTPC1_CLOPE - A0A023V983_CITFR - D5CDG9_ENTCC - T2A2Q7_STRAP - Q3Y285_ENTFC - T1ZZ00_STRAP - H6PCK0_STRIC - D5CEU5_ENTCC - A0A023V325_CITFR - D5CED2_ENTCC - D5CGF1_ENTCC - Q3XYX3_ENTFC - RPOC_PROAC - T2A3D5_STRAP - H6PAQ8_STRIC - D5CC00_ENTCC - V5VFT2_ACIBA - D5CDF0_ENTCC - A0A023NW04_9GAMM - G2SMP0_LACRR - T2A0N7_STRAP - H6PCJ8_STRIC - D5C883_ENTCC - H6PAQ5_STRIC - G2SR82_LACRR - Q3XYX5_ENTFC - H6PCL1_STRIC - Q3Y122_ENTFC - A0A023V7D7_CITFR - D5CCK4_ENTCC - D5CDF8_ENTCC - D5CDH3_ENTCC - RPOB_PROAC - D5CF33_ENTCC - D5C730_ENTCC - EFTU_PROAC - T2A1Q3_STRAP - A0A023V3X2_CITFR - D5C728_ENTCC - A0A023V7E0_CITFR - Q3XX08_ENTFC - D5CH16_ENTCC - R6XBD7_9PORP - T1ZZY5_STRAP - RS7_PROAC - H6PCJ5_STRIC - D5CF78_ENTCC - H6PCS3_STRIC - A0A095MA10_BURML - Hide IDs   **764**[active](https://www.uniprot.org/id-mapping/uniprotkb/604bb26250847a259c7af51aec5a8f91222e43e9/overview?query=active%3Atrue) entries and **119** [obsolete](https://www.uniprot.org/id-mapping/uniprotkb/604bb26250847a259c7af51aec5a8f91222e43e9/overview?query=active%3Afalse) entries are found |

Supplementary Table 5. Per species literature research on their prevalence and abundance in infant gut.

| **Species** | **Presence in infant gut based on literature** |
| --- | --- |
| *Bifidobacterium longum* subsp*. infantis* | 1^st^ most prevalent genus in bin numbers in 4-month vaginally born infants (Supplementary Fig. 1)  1^st^ most prevalent OTU at 4 months [23]  In the top most abundant genera in 1-month breastfed infants [24] |
| *Bifidobacterium breve* | 1^st^ most prevalent genus in bin numbers in 4-month vaginally born infants (Supplementary Fig. 1)  2^nd^ most prevalent OTU at 4 months [23]  In the top most abundant genera in 1-month breastfed infants [24] |
| *Bifidobacterium bifidum* | 1^st^ most prevalent genus in bin numbers in 4-month vaginally born infants (Supplementary Fig. 1)  3^rd^ most prevalent OTU at 4 months [23]  In the top most abundant genera in 1-month breastfed infants [24] |
| *Bacteroides fragilis* | 7^th^ most prevalent genus in bin numbers in 4-month vaginally born infants (Supplementary Fig. 1)  4^th^ most prevalent OTU at 4 months [23]  In the top most abundant genera in 1-month breastfed infants [24] |
| *Bacteroides vulgatus* | 3^rd^ most prevalent species in bin numbers in 4-month vaginally born infants (Supplementary Fig. 1)  In the top most abundant genera in 1-month breastfed infants [24]  5^th^ most prevalent OTU at 4 months [23] |
| *Ruminococcus gnavus* | 5^th^ most prevalent species in bin numbers in 4-month vaginally born infants (Supplementary Fig. 1)  9^th^ most prevalent OTU at 4 months [23]  High prevalence in infants [25] |
| *Bacteroides ovatus* | In the top most abundant genera in 1-month breastfed infants [24]  15^th^ most abundant OTU at 4 months [23]  13^th^ most prevalent species in bin numbers in 4-month vaginally born infants (Supplementary Fig. 1) |
| *Veillonella parvula* | 12^th^ most prevalent OTU at 4 months [23]  5^th^ most prevalent species in bin numbers in 4-month vaginally born infants (Supplementary Fig. 1)  In the top most abundant genera in 1-month breastfed infants [24] |
| *Escherichia coli* | 2^nd^ most prevalent species in bin numbers in 4-month vaginally born infants (Supplementary Fig. 1)  6^th^ most prevalent OTU at 4 months [23]  In the top most abundant genera in 1-month breastfed infants [24] |
| *Lactobacillus rhamnosus* | 18^th^ most prevalent OTU at 4 months [23]  12^th^ most prevalent species in bin numbers in 4-month vaginally born infants (Supplementary Fig. 1)  In the top most abundant genera in 1-month breastfed infants [61] |
| *Enterococcus faecalis* | 19^th^ most prevalent OTU at 4 months [23]  9th most prevalent species in bin numbers in 4-month vaginally born infants (Supplementary Fig. 1)  Characterized as the first infant colonizers [26] |
| *Streptococcus salivarus* | In the top most abundant genera in 1-month breastfed infants [24]  *Streptococcus* HGM10404 is the 10^th^ most prevalent species in bin numbers in 4-month vaginally born infants (Supplementary Fig. 1) but it is uncultured. The next is *Streptococcus salivarus*. |
| *Blautia producta* | *Blautia wexlerae* is the 22^th^ most prevalent species in bin numbers and only present in breastfed and mixed fed infants. However, Blautia producta is selected as it is better characterized and similarly possesses the Wood–Ljungdahl pathway.  Presence of *Blautia wexlerae/luti/producta* in infants aged 1 to 6 months [27] |


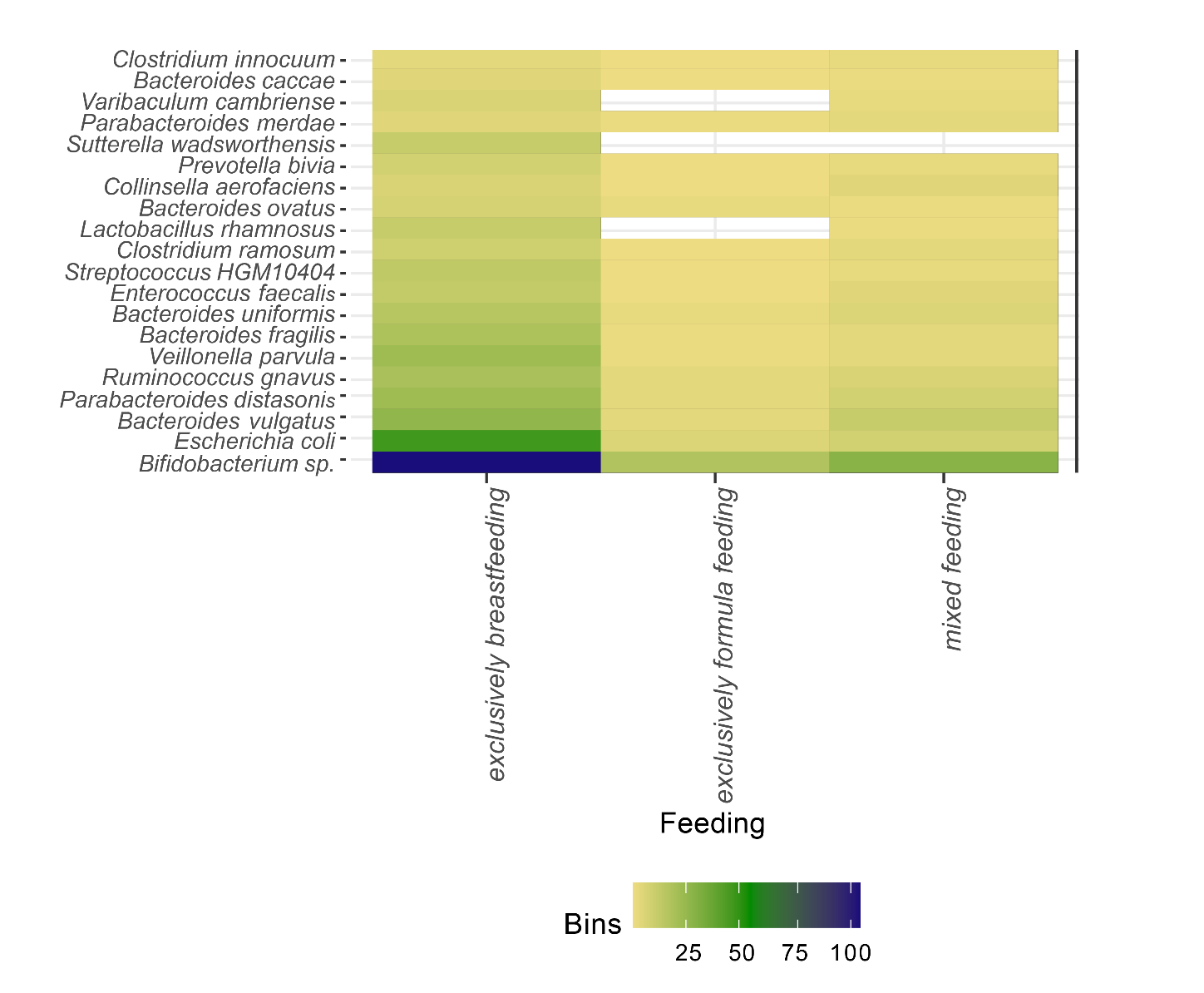


Supplementary Figure 1. Top 20 most prevalent (in terms of bin numbers across samples) species in 4-month vaginally born infants from publicly available MAGs [23, 28].


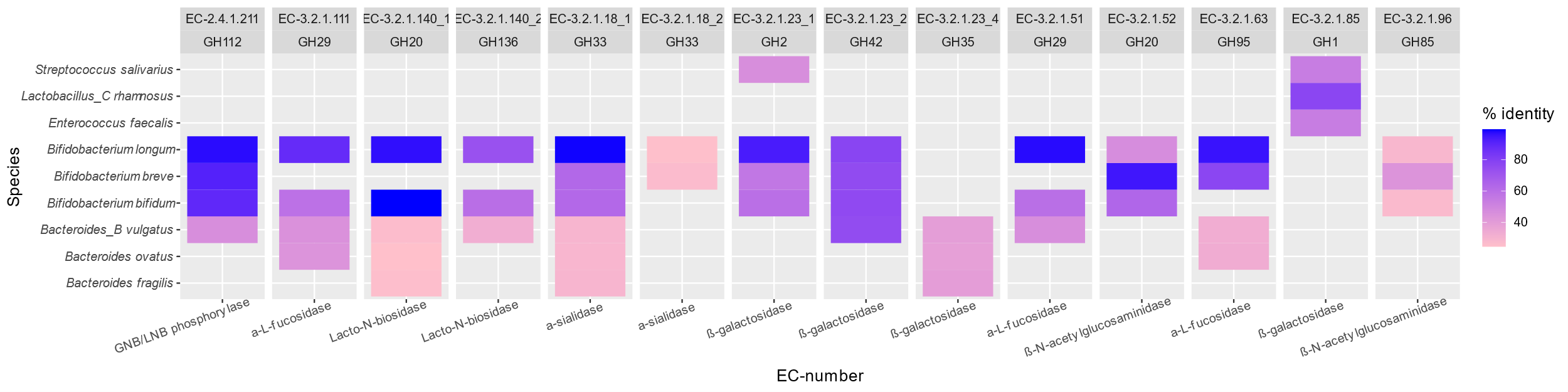


Supplementary Figure 2. Mean percentage identity of hits per relevant species against publicly available MAGs [23, 28] of infants aged 0, 4 and 12 months from various feeding and birth mode backgrounds.


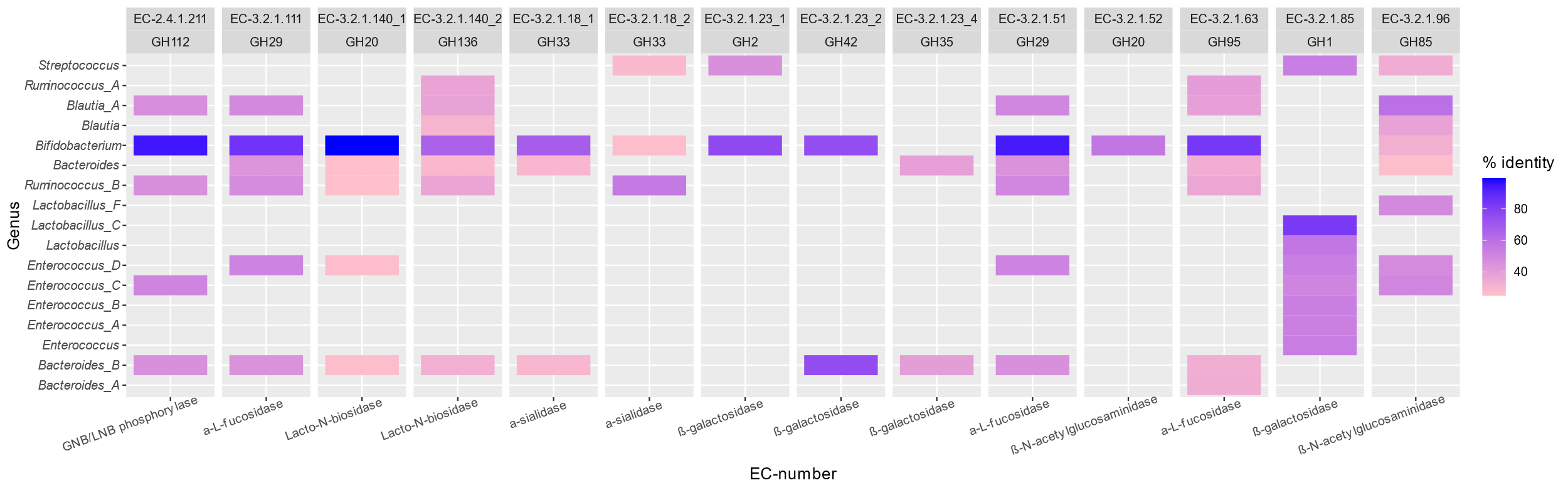


Supplementary Figure 3. Mean percentage identity of hits per genus against publicly available MAGs [23, 28] of infants aged 0, 4 and 12 months from various feeding and birth mode backgrounds.


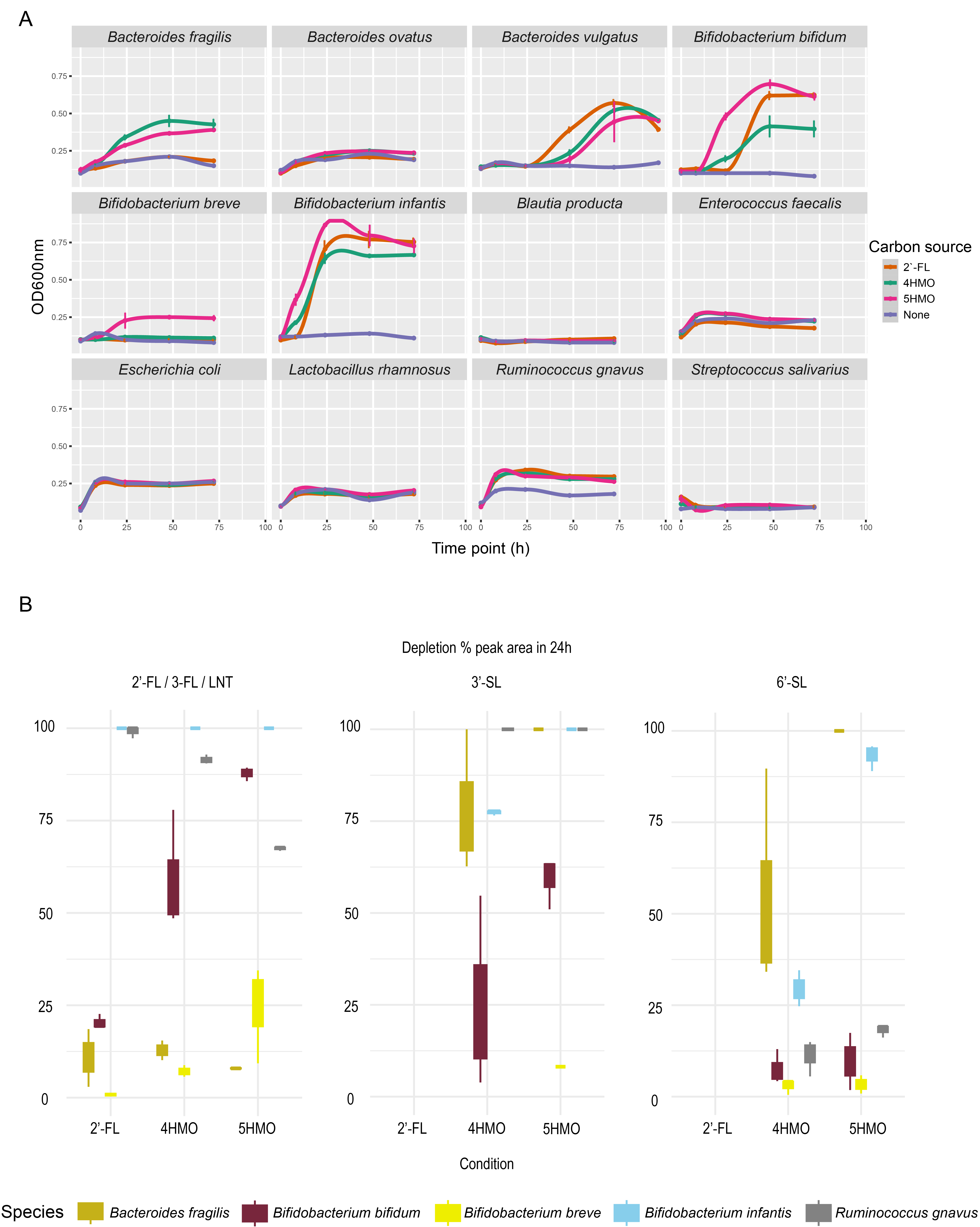


Supplementary Figure 4. A) Growth curves of BIG-Syc strains grown in 2’-FL, 4HMO mix, 5HMO mix and no carbon source, B) 2’-FL/3-FL/LNT, 3’-SL and 6’-SL as depletion % through utilization by HMO-degraders in the 2’-FL, 4HMO mix, and 5HMO mix as boxplot of three replicates.

Supplementary Table 7. Average purity (%) of community per experiment.

| **Condition** | **Relative abundance in % of community genera (Purity %)** |
| --- | --- |
| 4HMO 1 | 99.13 (SD **±** 0.80) |
| 4HMO 2 | 99.91 (SD **±** 0.17) |
| 5HMO | 99.93 (SD **±** 0.18) |
| 5HMO deletion/addition | 99.37 (SD **±** 1.17) |


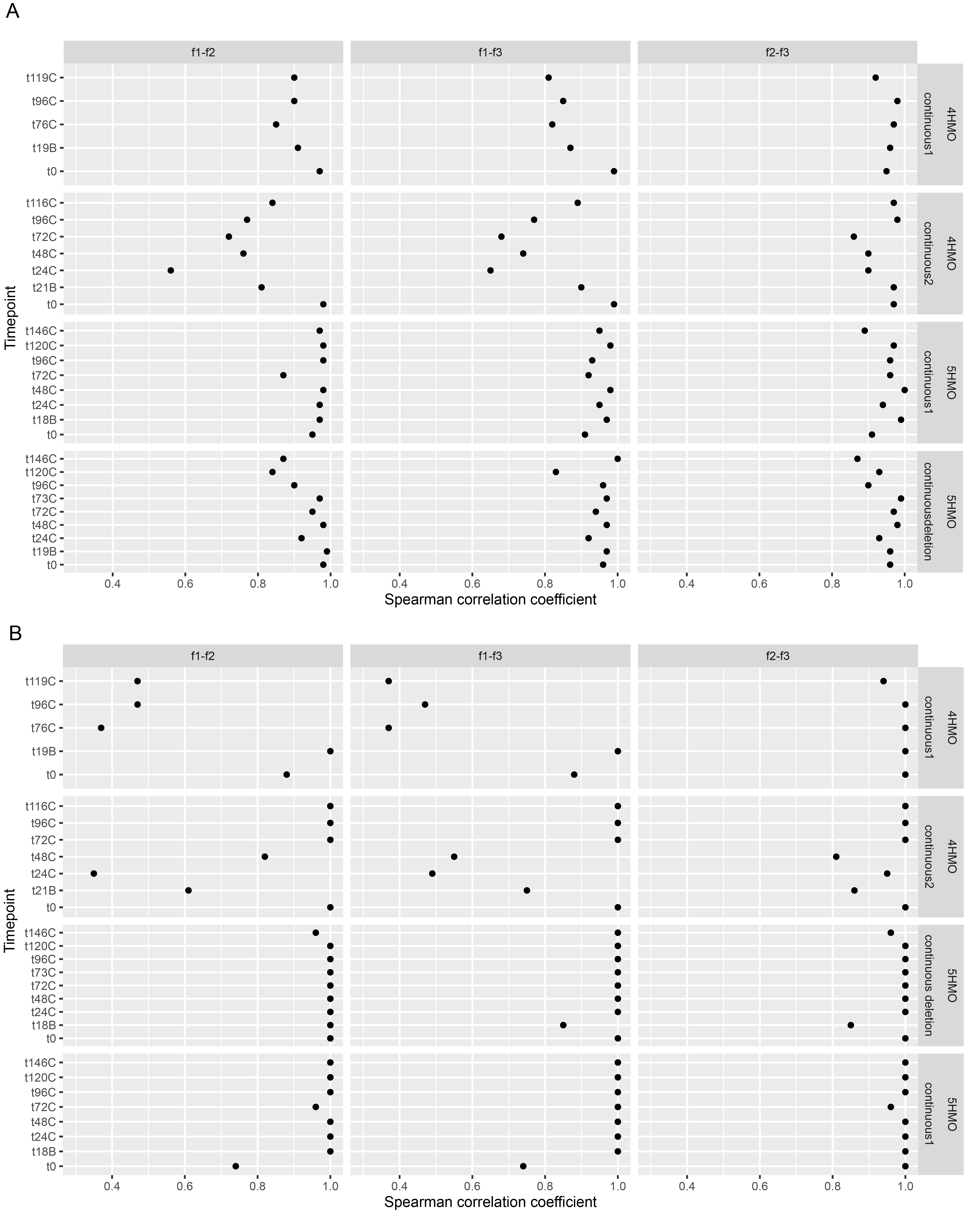


Supplementary Figure 5. Spearman correlation coefficient between fermentors per timepoint and grouped per experiment for A) composition profiles and B) metabolite profiles.


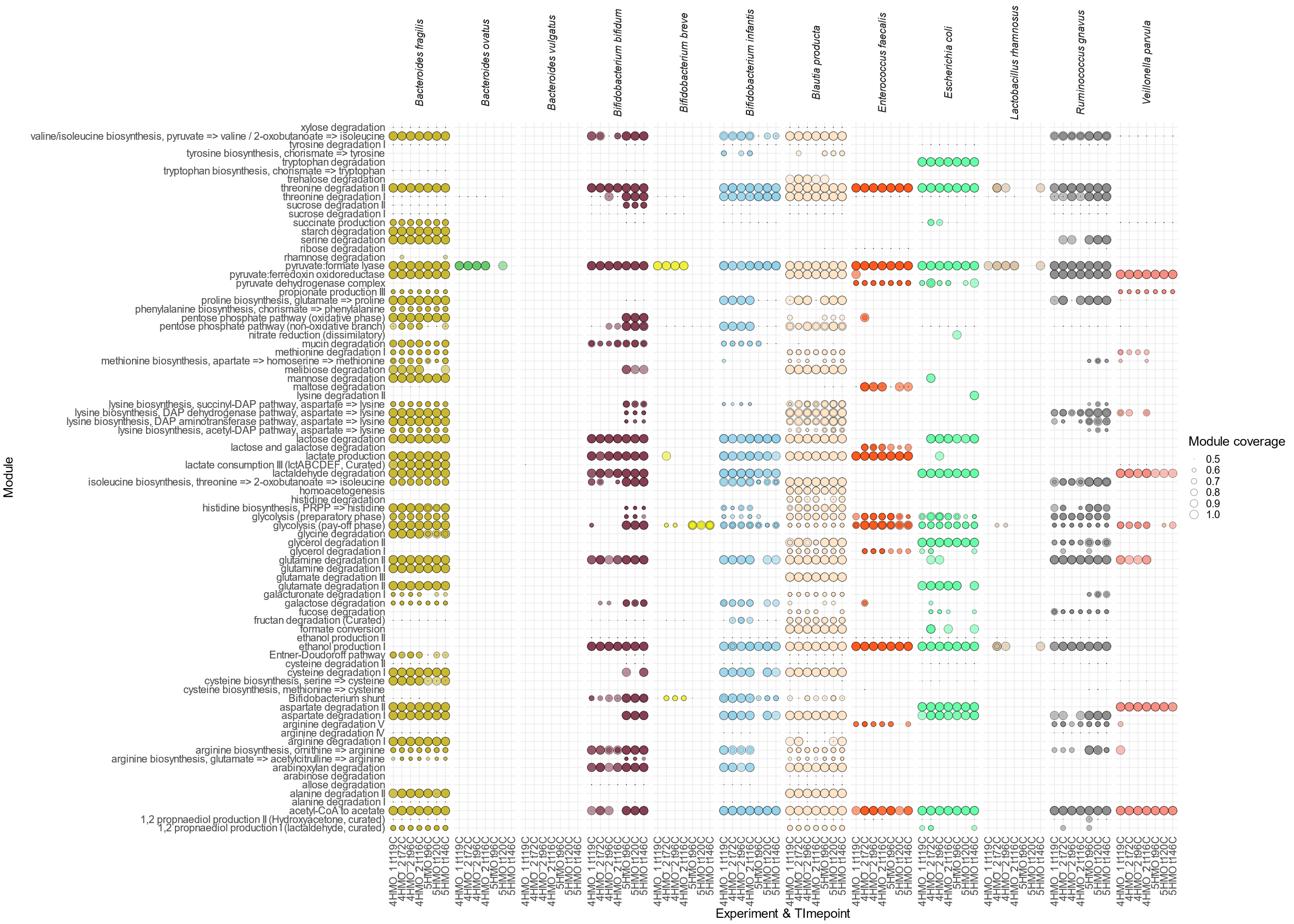


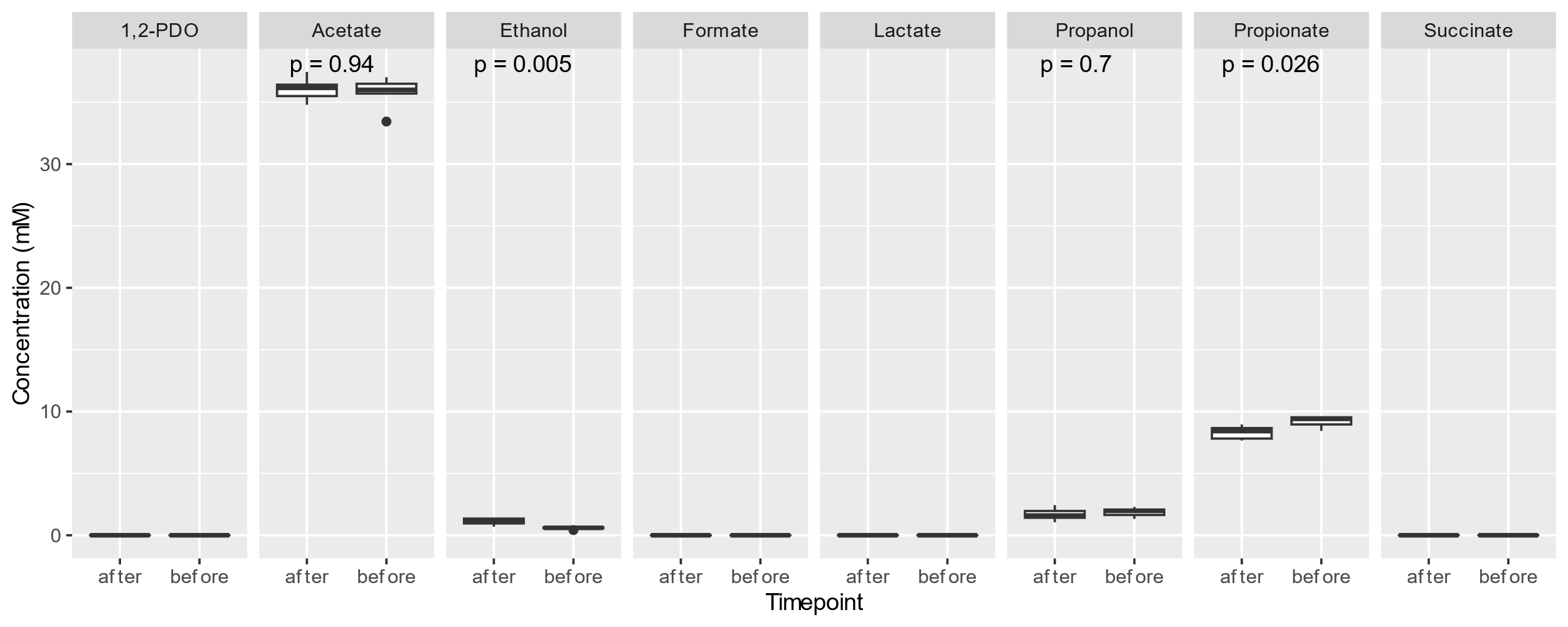
Supplementary Figure 6. Overview of the identified modules with >0.5 KO coverage in samples from the 4HMO 1, 4HMO 2, and 5HMO conditions. Values are grouped per module, experiment & timepoint, and species. Values from the three fermentors overlap. Lighter colour indicates the module was detected in fewer fermentors. Also, different coverages between fermentors lead to overlapping circles of different sizes.

Supplementary Figure 7. Mean concentration (mM) per compound before (t48C, t72C) and after (t120C, t146C) addition of bifidobacteria in the 5HMO deletion experiment with wilcoxon statistical testing *P* value.

Supplementary Table 9. Carbon sources utilized per species as justified by proteomics and monocultures.

| **Species** | **Input** | **Evidence** |
| --- | --- | --- |
| *Bifidobacterium infantis* | 2’-FL/3’-FL | Proteomics: fucosidase, beta-galactosidase, Blon_0342 & Blon_0343 FL transporter 1, Blon_2202 |
|  | LNT | Proteomics: endo-beta-N-acetylglucosaminidase, hexosaminidase, beta-galactosidase, GNB/LNB phosphorylase, Blon_2177 GltA LNT/LNB transporter |
|  | lactose | Proteomics: lactose degradation module |
|  | glucose | Proteomics: glycolysis module (preparatory and pay-off phase) |
|  | galactose | Proteomics: galactose degradation module  Galactose-1-phosphate uridylyltransferase |
|  | fucose | Proteomics: L-fucose mutarotase [EC:5.1.3.29] (conversion of alpha-L-Fucopyranose to beta-L-Fucopyranose), (conversion of beta-L-Fucopyranose to L-Fucono-1,5-lactone) |
|  | GlcNAc | Proteomics: N-acetylglucosamine-6-phosphate deacetylase [EC:3.5.1.25] (conversion of GlcNAc-6P to GlcN-6P), glucosamine-6-phosphate deaminase [EC:3.5.99.6] (conversion of GlcN-6P to Fru-6P), N-Acetyl-D-glucosamine 6-phosphate 2-epimerase [EC:5.1.3.9] (conversion of GlcNAc-6P to ManAc-6P |
| *Bifidobacterium bifidum* | 2’-FL/3’-FL | Proteomics: fucosidases, beta-galactosidases |
|  | 3’-SL/6’-SL | Proteomics: sialidase, beta-galactosidases |
|  | glucose | Proteomics: glycolysis module (preparatory and pay-off phase) |
|  | LNT | Proteomics: beta-N-acetylhexosaminidase, beta-galactosidase, hexosaminidase (EC 3.2.1.52) (which at UniProt is also annotated as Lacto-N-biosidase), BBPR_1056-1057 (GltABC homologues) that are responsible for LNB uptake |
|  | lactose | Proteomics: lactose degradation module  Beta-galactosidase |
|  | galactose | Proteomics: Galactose-1-phosphate uridylyltransferase (conversion of galactose to glucose) |
|  | GlcNAc | Proteomics: N-acetylglucosamine-6-phosphate deacetylase [EC:3.5.1.25] (conversion of GlcNAc-6P to GlcN-6P), glucosamine-6-phosphate deaminase [EC:3.5.99.6] (conversion of GlcN-6P to Fru-6P ) |
| *Bifidobacterium breve* | LNB | Proteomics: GNB/LNB phosphorylase, Bbr_1590 solute binding protein predicted GNB/LNB-specific part of the ABC transport system  Grows only in the 5HMO mix that contains LNT |
|  | lactose | Proteomics: lactose degradation module |
|  | glucose | Proteomics: complete pathway from glucose to pyruvate |
|  | GlcNAc | Proteomics: N-acetylglucosamine-6-phosphate deacetylase [EC:3.5.1.25] (conversion of GlcNAc-6P to GlcN-6P), glucosamine-6-phosphate deaminase [EC:3.5.99.6] (conversion of GlcN-6P to Fru-6P) and the rest of the pathway to pyruvate |
|  | galactose | Proteomics: Galactose-1-phosphate uridylyltransferase (conversion of galactose to glucose), all enzymes from D-galactose to α-D-Glucose-6P and α-D-Glucose |
| *Bacteroides fragilis* | 2’-FL/3’-FL | Proteomics: fucosidases, beta-galactosidases |
|  | 3’-SL/6’-SL | Proteomics: sialidases, beta-galactosidases |
|  | LNT | Proteomics: hexosaminidase [EC:3.2.1.52], beta-galactosidases |
|  | lactate | Proteomics: lactate consumption III module (including lactate permease for transport inside the cell). Restricted consumption evidence from literature[29]. More extensive consumption evidence [30]. In monoculture experiments, lactate is utilized after 384h in the fermentation (data not shown). |
|  | galactose | Proteomics: lactose degradation module, galactose degradation module |
|  | sialic acid | Proteomics: K01639 N-acetylneuraminate lyase [EC:4.1.3.3] |
|  | glucose | Proteomics: glycolysis (preparatory phase) module |
|  | fucose | Proteomics: L-fucose/D-arabinose isomerase [EC:5.3.1.25] (conversion of L-fucose to L-fuculose), GDP-L-fucose synthase [EC:1.1.1.271] (conversion of L-fucose to GDP-4-oxo-6-deoxy-D-manose) and two subsequent enzymes leading to manose-6P |
|  | GlcNAc | Proteomics: N-acetylglucosamine-6-phosphate deacetylase [EC:3.5.1.25] (conversion of GlcNAc-6P to GlcN-6P), glucosamine-6-phosphate deaminase [EC:3.5.99.6] (conversion of GlcN-6P to Fru-6P ) and the rest of the pathway to pyruvate |
| *Bacteroides vulgatus* | GlcNAc | Proteomics: expression of glucosamine-6-phosphate deaminase [EC:3.5.99.6] signifies conversion of GlcN-6P to Fru-6P and from then until Glycerate-3P with the enzymes: K00615 transketolase [EC:2.2.1.1]  K01803 triosephosphate isomerase (TIM)  K00134 glyceraldehyde 3-phosphate dehydrogenase  K00927 phosphoglycerate kinase [EC:2.7.2.3] |
| *Bacteroides ovatus* | glucose | Several enzymes from the glycolysis pathway are expressed. Also formate C-acetyltransferase is expressed which signifies presence of pyruvate. |
| *Veillonella parvula* | lactate | Growth in lactate as pre-culture  Proteomics: lactate consumption III module (including lactate permease for transport inside the cell) |
| *Escherichia coli* | lactose | Proteomics: lactose degradation module, beta-galactosidase production |
|  | galactose | Proteomics: galactose degradation module  Galactose-1-phosphate uridylyltransferase |
|  | fucose | Proteomics: fucose degradation module |
|  | glucose | Proteomics: glycolysis (preparatory phase) module |
|  | GlcNAc | Proteomics: multiple PTS system proteins expressed which are responsible for the uptake in other species [31], N-acetylglucosamine-6-phosphate deacetylase [EC:3.5.1.25] (conversion of GlcNAc-6P to GlcN-6P), glucosamine-6-phosphate deaminase [EC:3.5.99.6] signifies conversion of GlcN-6P to Fru-6P, N-Acetyl-D-glucosamine 6-phosphate 2-epimerase [EC:5.1.3.9] (conversion of GlcNAc-6P to ManAc-6P |
|  | sialic acid | Proteomics: K01639 N-Acetylneuraminate (pyruvate-)lyase [EC:4.1.3.3]  K17948 N-acetylneuraminate epimerase [EC:5.1.3.24] |
| *Blautia producta* | lactose | Proteomics: lactose degradation module |
|  | galactose | Proteomics: galactose degradation module  Galactose-1-phosphate uridylyltransferase |
|  | fucose | Proteomics: fucose degradation module |
|  | glucose | Proteomics: glycolysis (preparatory phase) module |
|  | sialic acid | Proteomics: K01639 N-acetylneuraminate lyase [EC:4.1.3.3] |
|  | H_2_ & CO_2_ | Proteomics: complete Wood-Ljungdahl pathway   \| [K00198](https://www.genome.jp/entry/K00198): cooS, acsA; anaerobic carbon-monoxide dehydrogenase catalytic subunit [EC:[1.2.7.4](https://www.genome.jp/entry/1.2.7.4)] \| \| --- \| \| [K05299](https://www.genome.jp/entry/K05299): fdhA; formate dehydrogenase (NADP+) alpha subunit [EC:[1.17.1.10](https://www.genome.jp/entry/1.17.1.10)] \| \| [K01938](https://www.genome.jp/entry/K01938): fhs; formate--tetrahydrofolate ligase [EC:[6.3.4.3](https://www.genome.jp/entry/6.3.4.3)] \| \| [K01500](https://www.genome.jp/entry/K01500): fchA; methenyltetrahydrofolate cyclohydrolase [EC:[3.5.4.9](https://www.genome.jp/entry/3.5.4.9)] \| \| [K00297](https://www.genome.jp/entry/K00297): metF, MTHFR; methylenetetrahydrofolate reductase (NADH) [EC:[1.5.1.54](https://www.genome.jp/entry/1.5.1.54)] \| \| [K15023](https://www.genome.jp/entry/K15023): acsE; 5-methyltetrahydrofolate corrinoid/iron sulfur protein methyltransferase [EC:[2.1.1.258](https://www.genome.jp/entry/2.1.1.258)] \| \| [K14138](https://www.genome.jp/entry/K14138): acsB; acetyl-CoA synthase [EC:[2.3.1.169](https://www.genome.jp/entry/2.3.1.169)] \| \| [K00197](https://www.genome.jp/entry/K00197): cdhE, acsC; acetyl-CoA decarbonylase/synthase, CODH/ACS complex subunit gamma [EC:[2.1.1.245](https://www.genome.jp/entry/2.1.1.245)] \| \| [K00194](https://www.genome.jp/entry/K00194): cdhD, acsD; acetyl-CoA decarbonylase/synthase, CODH/ACS complex subunit delta [EC:[2.1.1.245](https://www.genome.jp/entry/2.1.1.245)] \| |
|  | GlcNAc | Proteomics: multiple PTS system proteins expressed which are responsible for the uptake in other species [31], N-acetylglucosamine-6-phosphate deacetylase [EC:3.5.1.25] (conversion of GlcNAc-6P to GlcN-6P), glucosamine-6-phosphate deaminase [EC:3.5.99.6] signifies conversion of GlcN-6P to Fru-6P, N-Acetyl-D-glucosamine 6-phosphate 2-epimerase [EC:5.1.3.9] (conversion of GlcNAc-6P to ManAc-6P |
| *Enterococcus faecalis* | lactose | Proteomics: lactose and galactose degradation module,  Beta-galactosidase |
|  | glucose | Proteomics: complete pathway from glucose to pyruvate |
|  | GlcNAc | Proteomics: multiple PTS system proteins expressed which are responsible for the uptake in other species [31], N-acetylglucosamine-6-phosphate deacetylase [EC:3.5.1.25] (conversion of GlcNAc-6P to GlcN-6P), glucosamine-6-phosphate deaminase [EC:3.5.99.6] signifies conversion of GlcN-6P to Fru-6P, N-Acetyl-D-glucosamine 6-phosphate 2-epimerase [EC:5.1.3.9] (conversion of GlcNAc-6P to ManAc-6P |
|  | galactose | Proteomics: Galactose-1-phosphate uridylyltransferase, lactose and galactose degradation pathway |
| *Lactobacillus rhamnosus* | glucose | Proteomics: majority of enzymes from glucose to pyruvate |
|  | fucose | Proteomics: L-fucose/D-arabinose isomerase [EC:5.3.1.25] (conversion of L-fucose to L-fuculose) |
| *Ruminococcus gnavus* | LNB | Proteomics: GNB/LNB phosphorylase [EC:2.4.1.211] |
|  | 2’-FL/3’-FL | Proteomics: fucosidases |
|  | 3’-SL/6’-SL | Proteomics: endosialidase  Degradation, nevertheless, small concentragtions, in in-house experiments (data not shown) |
|  | fucose | Proteomics: fucose degradation module |
|  | glucose | Proteomics: glycolysis (preparatory phase) module |
|  | galactose | Proteomics: Galactose-1-phosphate uridylyltransferase |
|  | GlcNAc | Proteomics: multiple PTS system proteins expressed which are responsible for the uptake in other species [31], N-acetylglucosamine-6-phosphate deacetylase [EC:3.5.1.25] (conversion of GlcNAc-6P to GlcN-6P), glucosamine-6-phosphate deaminase [EC:3.5.99.6] signifies conversion of GlcN-6P to Fru-6P, N-Acetyl-D-glucosamine 6-phosphate 2-epimerase [EC:5.1.3.9] (conversion of GlcNAc-6P to ManAc-6P |
|  | sialic acid | Proteomics: K01639 N-acetylneuraminate lyase [EC:4.1.3.3] |
| *Streptococcus salivarius* | glucose | Proteomics: various enzymes from the glycolysis pathway |

Supplementary Table 10. Metabolites and gases produced per species as justified by proteomics, monocultures and metabolic modelling analysis.

| **Species** | **Output** | **Evidence** |
| --- | --- | --- |
| *Ruminococcus*  *gnavus* | Lactose | Proteomics: fucosidases but no beta-galactosidases  Detected in monoculture (data not shown) |
|  | 1,2-PDO | Proteomics: 1,2 propanediol production I/II modules |
|  | 1-propanol | Proteomics: K01699 propanediol dehydratase large (conversion of 1,2-PDO to propanal), K13921 1-propanol dehydrogenase (conversion of propanal to 1-propanol) |
|  | Propionate | Detected in monoculture (data not shown)  Proteomics: K01699 propanediol dehydratase large EC 4.2.1.28 (1,2-PDO to propanal); K13922 propionaldehyde dehydrogenase EC 1.2.1.87 (propanal to propanoyl-CoA). |
|  | acetate | Detected in monoculture (data not shown)  Proteomics: K00625 phosphate acetyltransferase (acetyl-CoA to acetate); K15024 putative phosphotransacetylase EC 2.3.1.8 (acetyl-CoA to acetate) ; K00925 acetate kinase EC 2.7.2.1 (acetyl-CoA to acetate) |
|  | formate | Detected in monoculture (data not shown)  Proteomics: K04069 pyruvate formate lyase (pyruvate + CoA ⇌ acetyl-CoA + formate); K00656 formate C-acetyltransferase EC 2.3.1.54 (pyruvate + CoA ⇌ acetyl-CoA + formate); |
|  | succinate | Detected in monoculture (data not shown) |
|  | ethanol | Proteomics: ethanol production I/II module |
|  | CO_2_ | Proteomics: phosphoenolpyruvate carboxykinase, pyruvate-ferredoxin/flavodoxin oxidoreductase, acetolactate synthase I/II/III large subunit, acetolactate synthase I/III small subunit, isocitrate dehydrogenase, prephenate dehydratase  Predicted production via Genome Scale Metabolic Models |
| *Bifidobacterium infantis* | acetate | Detected in monoculture (data not shown)  Proteomics: K13788 phosphate acetyltransferase EC 2.3.1.8 (acetyl-CoA to acetate) ; K00925 acetate kinase EC 2.7.2.1 (acetyl-CoA to acetate) |
|  | formate | Detected in monoculture (data not shown)  Proteomics: K04069 pyruvate formate lyase (pyruvate + CoA ⇌ acetyl-CoA + formate); K00656 formate C-acetyltransferase EC 2.3.1.54 (pyruvate + CoA ⇌ acetyl-CoA + formate); |
|  | succinate | Proteomics: succinate production module |
|  | lactate | Detected in monoculture (data not shown)  Proteomics: lactate production module |
|  | ethanol | Proteomics: ethanol production I/II module |
|  | CO_2_ | Proteomics: dihydrolipoyl dehydrogenase, phosphoenolpyruvate carboxylase, acetolactate synthase I/II/III large subunit, acetolactate synthase I/III small subunit, carbonic anhydrase, isocitrate dehydrogenase  Predicted production via Genome Scale Metabolic Models |
| *Bifidobacterium bifidum* | acetate | Detected in monoculture (data not shown)  Proteomics: K13788 phosphate acetyltransferase EC 2.3.1.8 (acetyl-CoA to acetate); K00925 acetate kinase EC 2.7.2.1 (acetyl-CoA to acetate) |
|  | formate | Detected in monoculture (data not shown)  Proteomics: K04069 pyruvate formate lyase (pyruvate + CoA ⇌ acetyl-CoA + formate); K00656 formate C-acetyltransferase EC 2.3.1.54 (pyruvate + CoA ⇌ acetyl-CoA + formate); |
|  | succinate | Proteomics: succinate production module |
|  | lactate | Detected in monoculture (data not shown)  Proteomics: lactate production module |
|  | ethanol | Proteomics: ethanol production I/II module |
|  | CO_2_ | Proteomics: phosphoenolpyruvate carboxylase, acetolactate synthase I/II/III large subunit, acetolactate synthase I/III small subunit, carbonic anhydrase, isocitrate dehydrogenase  Predicted production via Genome Scale Metabolic Models |
| *Bifidobacterium breve* | acetate | Detected in monoculture (data not shown)  Proteomics: K13788 phosphate acetyltransferase EC 2.3.1.8 (acetyl-CoA to acetate). |
|  | formate | Detected in monoculture (data not shown)  Proteomics: K00656 formate C-acetyltransferase EC 2.3.1.54 (pyruvate + CoA ⇌ acetyl-CoA + formate); |
|  | lactate | Detected in monoculture (data not shown)  Proteomics: lactate production module |
|  | CO_2_ | Proteomics: phosphoenolpyruvate carboxylase, acetolactate synthase I/II/III large subunit  Predicted production via Genome Scale Metabolic Models |
| *Blautia producta* | acetate | Proteomics: K15024 putative phosphotransacetylase EC 2.3.1.8 (acetyl-CoA to acetate) ; K00925 acetate kinase EC 2.7.2.1 (acetyl-CoA to acetate) |
|  | formate | Proteomics: K04069 pyruvate formate lyase (pyruvate + CoA ⇌ acetyl-CoA + formate); K00656 formate C-acetyltransferase EC 2.3.1.54 (pyruvate + CoA ⇌ acetyl-CoA + formate); |
|  | lactate | Proteomics: lactate production module |
|  | ethanol | Proteomics: ethanol production I/II module |
|  | 1,2-PDO | Proteomics: 1,2 propanediol production II modules |
|  | CO_2_ | Proteomics: phosphoenolpyruvate carboxykinase, pyruvate-ferredoxin / flavodoxin oxidoreductase, acetolactate synthase I/II/III large subunit, acetolactate synthase I/III small subunit, isocitrate dehydrogenase, prephenate dehydratase (enzymes from the Wood-Ljungdahl pathway are not included)  Predicted production via Genome Scale Metabolic Models and measured when the strain is grown in basal medium with lactose as carbon source (data not shown) |
|  | H_2_ | Proteomics: For H^+^ production: Glycolysis pathway, 2-oxoglutarate/2-oxoacid ferredoxin oxidoreductase subunit, pyruvate-ferredoxin/flavodoxin oxidoreductase, glyceraldehyde 3-phosphate dehydrogenase (enzymes from the Wood-Ljungdahl pathway are not included)  For H_2_ from H^+^ and e^-^:  F420-non-reducing hydrogenase iron-sulfur, heterodisulfide reductase subunit A2  Measured when the strain is grown in basal medium with lactose as carbon source (data not shown) |
| *Escherichia coli* | acetate | Proteomics: K13788 phosphate acetyltransferase EC 2.3.1.8 (acetyl-CoA to acetate) ; K00925 acetate kinase EC 2.7.2.1 (acetyl-CoA to acetate) |
|  | formate | Proteomics: K00656 formate C-acetyltransferase EC 2.3.1.54 (pyruvate + CoA ⇌ acetyl-CoA + formate); K04069 pyruvate formate lyase (pyruvate + CoA ⇌ acetyl-CoA + formate); K06212 formate transporter, |
|  | succinate | Proteomics: succinate production module |
|  | lactate | Proteomics: lactate production module |
|  | ethanol | Proteomics: ethanol production I/II module |
|  | 1,2-PDO | Proteomics: 1,2 propanediol production I/II modules |
|  | CO_2_ | Proteomics: formate dehydrogenase, Pyruvate dehydrogenase E1, dihydrolipoyl dehydrogenase, phosphoenolpyruvate carboxykinase, glycine dehydrogenase,  malate dehydrogenase, isocitrate dehydrogenase, 3-hydroxy acid dehydrogenase  Predicted production via Genome Scale Metabolic Models |
|  | H_2_ | Proteomics: For H^+^ production: Glycolysis, glycine dehydrogenase, malate dehydrogenase  For H_2_ from H^+^ and e^-^:  hydrogenase large subunit, hydrogenase small subunit, formate dehydrogenase (hydrogenase)  Measured when the strain is grown in LB medium (data not shown) |
| *Enterococcus faecalis* | acetate | Proteomics: K13788 phosphate acetyltransferase EC 2.3.1.8 (acetyl-CoA to acetate) ; K00925 acetate kinase EC 2.7.2.1 (acetyl-CoA to acetate) |
|  | formate | Proteomics: K00656 formate C-acetyltransferase EC 2.3.1.54 (pyruvate + CoA ⇌ acetyl-CoA + formate); K04069 pyruvate formate lyase (pyruvate + CoA ⇌ acetyl-CoA + formate); |
|  | lactate | Proteomics: lactate production module |
|  | ethanol | Proteomics: ethanol production II module |
|  | 1,2-PDO | Proteomics: 1,2 propanediol production I/II modules |
|  | CO_2_ | Proteomics: pyruvate dehydrogenase, dihydrolipoyl dehydrogenase, pyruvate-ferredoxin/flavodoxin oxidoreductase  Predicted production via Genome Scale Metabolic Models |
| *Veilonella parvula* | acetate | Proteomics: K13788 phosphate acetyltransferase EC 2.3.1.8 (acetyl-CoA to acetate) ; K00925 acetate kinase EC 2.7.2.1 (acetyl-CoA to acetate) |
|  | formate | Proteomics: Proteomics: K00656 formate C-acetyltransferase EC 2.3.1.54 (pyruvate + CoA ⇌ acetyl-CoA + formate); |
|  | succinate | Proteomics: succinate production module |
|  | propionate | Proteomics: propionate production III module (succinate pathway) |
|  | CO_2_ | Proteomics: pyruvate dehydrogenase, phosphoenolpyruvate carboxykinase, pyruvate-ferredoxin/flavodoxin oxidoreductase, acetolactate synthase I/II/III large subunit, acetolactate synthase I/III small subunit, prephenate dehydratase |
|  | H_2_ | Proteomics: For H^+^ production: glycolysis, 2-oxoglutarate/2-oxoacid ferredoxin oxidoreductase subunit, pyruvate:ferredoxin 2-oxidoreductase, pyruvate-ferredoxin/flavodoxin oxidoreductase  For H_2_ from H^+^ and e^-^:  hydrogenase large subunit, hydrogenase small subunit, heterodisulfide reductase subunit B2 |
| *Bacteroides fragilis* | acetate | Detected in monoculture (data not shown)  Proteomics: K13788 phosphate acetyltransferase EC 2.3.1.8 (acetyl-CoA to acetate) ; K00925 acetate kinase EC 2.7.2.1 (acetyl-CoA to acetate) |
|  | propionate | Proteomics: propionate production III module (succinate pathway) |
|  | formate | Proteomics: K00656 formate C-acetyltransferase EC 2.3.1.54 (pyruvate + CoA ⇌ acetyl-CoA + formate); K04069 pyruvate formate lyase (pyruvate + CoA ⇌ acetyl-CoA + formate); K06212 formate transporter |
|  | succinate | Detected in monoculture (data not shown)  Proteomics: succinate production module |
|  | lactate | Detected in monoculture (data not shown)  Proteomics: lactate production module |
|  | 1,2-PDO | Proteomics: 1,2 propanediol production I/II modules |
|  | CO_2_ | Proteomics: pyruvate dehydrogenase, dihydrolipoyl dehydrogenase, phosphoenolpyruvate carboxykinase, glycine dehydrogenase, malate dehydrogenase, pyruvate-ferredoxin/flavodoxin oxidoreductase, acetolactate synthase I/II/III large subunit, acetolactate synthase I/III small subunit, carbonic anhydrase, isocitrate dehydrogenase, prephenate dehydratase, 3-hydroxy acid dehydrogenase |
| *Bacteroides vulgatus* | acetate | Proteomics: K00925 acetate kinase EC 2.7.2.1  Detected in monoculture with minimal medium and HMOs (data not shown) |
|  | propionate | Proteomics: K00925 acetate kinase EC 2.7.2.1  KEGG (https://www.genome.jp/entry/K00925+2.7.2.1+R01353) as well as BioCyc (<https://biocyc.org/gene?orgid=ECOLI&id=ACETATEKINA-MONOMER> entrly for *E. coli*) show that this acetate kinase has also propionate kinase activity.  Detected in monoculture with minimal medium and HMOs (data not shown) |
|  | succinate | Proteomics: K00239 succinate dehydrogenase flavoprotein  Detected in monoculture with minimal medium and HMOs (data not shown) |
|  | CO_2_ | Proteomics: phosphoenolpyruvate carboxykinase, 2-oxoglutarate/2-oxoacid ferredoxin oxidoreductase |
| *Lactobacillus rhamnosus* | formate | Proteomics: K00656 formate C-acetyltransferase EC 2.3.1.54 (pyruvate + CoA ⇌ acetyl-CoA + formate); |
|  | ethanol | Proteomics: ethanol production I/II module |
| *Bacteroides ovatus* | formate | Proteomics: K00656 formate C-acetyltransferase EC 2.3.1.54 (pyruvate + CoA ⇌ acetyl-CoA + formate); |
|  | CO_2_ | Proteomics: prephenate dehydratase, 3-hydroxy acid dehydrogenase |

**References**

1. Ioannou A, Knol J, Belzer C. Microbial glycoside hydrolases in the first year of life: an analysis review on their presence and importance in infant gut. *Front Microbiol* 2021; 12: 631282.

2. Arzamasov AA, Osterman AL. Milk glycan metabolism by intestinal bifidobacteria: insights from comparative genomics. *Crit Rev Biochem Mol Biol* 2022; 57: 562–584.

3. Arzamasov AA, Nakajima A, Sakanaka M, Ojima MN, Katayama T, Rodionov DA, et al. Human milk oligosaccharide utilization in intestinal bifidobacteria Is governed by global transcriptional regulator NagR. *mSystems* 2022; 7: e00343-22.

4. Zabel BE, Gerdes S, Evans KC, Nedveck D, Singles SK, Volk B, et al. Strain-specific strategies of 2′-fucosyllactose, 3-fucosyllactose, and difucosyllactose assimilation by *Bifidobacterium longum* subsp. *infantis* Bi-26 and ATCC 15697. *Sci Rep* 2020; 10: 15919.

5. Asakuma S, Hatakeyama E, Urashima T, Yoshida E, Katayama T, Yamamoto K, et al. Physiology of consumption of human milk oligosaccharides by infant gut-associated *Bifidobacteria*. *J Biol Chem* 2011; 286: 34583–34592.

6. Schöpping M, Zeidan AA, Franzén CJ. Stress Response in Bifidobacteria. *Microbiol Mol Biol Rev* 2022; 86: e00170-21.

7. Duncan SH, Louis P, Thomson JM, Flint HJ. The role of pH in determining the species composition of the human colonic microbiota. *Environ Microbiol* 2009; 11: 2112–2122.

8. Khandelwal RA, Olivier BG, Röling WFM, Teusink B, Bruggeman FJ. Community flux balance analysis for microbial consortia at balanced growth. *PLoS One* 2013; 8: e64567.

9. Chan SHJ, Simons MN, Maranas CD. SteadyCom: Predicting microbial abundances while ensuring community stability. *PLoS Comput Biol* 2017; 13: e1005539.

10. Diener C, Gibbons SM, Resendis-Antonio O. MICOM: Metagenome-scale modeling to infer metabolic interactions in the gut microbiota. *mSystems* 2020; 5: e00606-19.

11. Scott WT, Benito-Vaquerizo S, Zimmermann J, Bajić D, Heinken A, Suarez-Diez M, et al. A structured evaluation of genome-scale constraint-based modeling tools for microbial consortia. *PLoS Comput Biol* 2023; 19: e1011363.

12. Versluis DM, Wijtkamp C, Looijesteijn E, Geurts JMW, Merks RMH. Simulations of the infant gut microbiota suggest that complex ecological interactions regulate effects of human milk oligosaccharides on microbial mucin consumption. *bioRxiv* 2024; 2024.07.15.603541.

13. Espey MG. Role of oxygen gradients in shaping redox relationships between the human intestine and its microbiota. *Free Radic Biol Med* 2013; 55: 130–140.

14. Garrido D, Dallas DC, Mills DA. Consumption of human milk glycoconjugates by infant-associated bifidobacteria: mechanisms and implications. *Microbiology (N Y)* 2013; 159: 649–664.

15. Gotoh A, Katoh T, Sakanaka M, Ling Y, Yamada C, Asakuma S, et al. Sharing of human milk oligosaccharides degradants within bifidobacterial communities in faecal cultures supplemented with *Bifidobacterium bifidum*. *Sci Rep* 2018; 8: 13958.

16. Monk JM, Koza A, Campodonico MA, Machado D, Seoane JM, Palsson BO, et al. Multi-omics quantification of species variation of *Escherichia coli* links molecular features with strain phenotypes. *Cell Syst* 2016; 3: 238-251.e12.

17. Lam TJ, Stamboulian M, Han W, Ye Y. Model-based and phylogenetically adjusted quantification of metabolic interaction between microbial species. *PLoS Comput Biol* 2020; 16: e1007951.

18. Basile A, Campanaro S, Kovalovszki A, Zampieri G, Rossi A, Angelidaki I, et al. Revealing metabolic mechanisms of interaction in the anaerobic digestion microbiome by flux balance analysis. *Metab Eng* 2020; 62: 138–149.

19. da Silva VG, Smith NW, Mullaney JA, Wall C, Roy NC, McNabb WC. Food-breastmilk combinations alter the colonic microbiome of weaning infants: an *in silico* study. *mSystems* 2024; 9.

20. McGuire MK, Meehan CL, McGuire MA, Williams JE, Foster J, Sellen DW, et al. What’s normal? Oligosaccharide concentrations and profiles in milk produced by healthy women vary geographically. *Am J of Clin Nutr* 2017; 105: 1086–1100.

21. Walker AW, Martin JC, Scott P, Parkhill J, Flint HJ, Scott KP. 16S rRNA gene-based profiling of the human infant gut microbiota is strongly influenced by sample processing and PCR primer choice. *Microbiome* 2015; 3: 26.

22. Martin R, Makino H, Yavuz AC, Ben-Amor K, Roelofs M, Ishikawa E, et al. Early-life events, including mode of delivery and type of feeding, siblings and gender, shape the developing gut microbiota. *PLoS One* 2016; 11: e0158498.

23. Bäckhed F, Roswall J, Peng Y, Feng Q, Jia H, Kovatcheva-Datchary P, et al. Dynamics and stabilization of the human gut microbiome during the first year of life. *Cell Host Microbe* 2015; 17: 690–703.

24. Borewicz K, Gu F, Saccenti E, Arts ICW, Penders J, Thijs C, et al. Correlating infant fecal microbiota composition and human milk oligosaccharide consumption by microbiota of 1‐month‐old breastfed infants. *Mol Nutr Food Res* 2019; 63: 1801214.

25. Sagheddu V, Patrone V, Miragoli F, Puglisi E, Morelli L. Infant early gut colonization by Lachnospiraceae: high frequency of *Ruminococcus gnavus*. *Front Pediatr* 2016; 4: 1.

26. Adlerberth I, Wold AE. Establishment of the gut microbiota in Western infants. *Acta Paediatrica, Int J Paediatr* 2009. John Wiley & Sons, Ltd. , 98: 229–238

27. Sagheddu V, Patrone V, Miragoli F, Morelli L. Abundance and diversity of hydrogenotrophic microorganisms in the infant gut before the weaning period sssessed by Denaturing Gradient Gel Electrophoresis and quantitative PCR. *Front Nutr* 2017; 4: 26.

28. Nayfach S, Shi ZJ, Seshadri R, Pollard KS, Kyrpides NC. New insights from uncultivated genomes of the global human gut microbiome. *Nature* 2019; 568: 505–510.

29. Rios-Covian D, Arboleya S, Hernandez-Barranco AM, Alvarez-Buylla JR, Ruas-Madiedo P, Gueimonde M, et al. Interactions between *Bifidobacterium* and *Bacteroides* species in cofermentations are affected by carbon sources, including exopolysaccharides produced by Bifidobacteria. *Appl Environ Microbiol* 2013; 79: 7518–7524.

30. Macy JM, Ljungdahl LG, Gottschalk G. Pathway of succinate and propionate formation in *Bacteroides fragilis*. *J Bacteriol* 1978; 134: 84–91.

31. Garrido D, Nwosu C, Ruiz-Moyano S, Aldredge D, German JB, Lebrilla CB, et al. Endo-β-N-acetylglucosaminidases from infant gut-associated bifidobacteria release complex N-glycans from human milk glycoproteins. *Mol Cell Proteom* 2012; 11: 775–785.
